# Controlling Dengue with Vaccines in Thailand

**DOI:** 10.1371/journal.pntd.0001876

**Published:** 2012-10-25

**Authors:** Dennis L. Chao, Scott B. Halstead, M. Elizabeth Halloran, Ira M. Longini

**Affiliations:** 1 Center for Statistics and Quantitative Infectious Diseases, Vaccine and Infectious Disease Division, Fred Hutchinson Cancer Research Center, Seattle, Washington, United States of America; 2 Dengue Vaccine Initiative, Seoul, South Korea; 3 Department of Biostatistics, School of Public Health, University of Washington, Seattle, Washington, United States of America; 4 Department of Biostatistics, College of Public Health and Health Professions, and Emerging Pathogens Institute, University of Florida, Gainesville, Florida, United States of America; Yale University, United States of America

## Abstract

**Background:**

Dengue is a mosquito-borne infectious disease that constitutes a growing global threat with the habitat expansion of its vectors *Aedes aegyti* and *A. albopictus* and increasing urbanization. With no effective treatment and limited success of vector control, dengue vaccines constitute the best control measure for the foreseeable future. With four interacting dengue serotypes, the development of an effective vaccine has been a challenge. Several dengue vaccine candidates are currently being tested in clinical trials. Before the widespread introduction of a new dengue vaccine, one needs to consider how best to use limited supplies of vaccine given the complex dengue transmission dynamics and the immunological interaction among the four dengue serotypes.

**Methodology/Principal Findings:**

We developed an individual-level (including both humans and mosquitoes), stochastic simulation model for dengue transmission and control in a semi-rural area in Thailand. We calibrated the model to dengue serotype-specific infection, illness and hospitalization data from Thailand. Our simulations show that a realistic roll-out plan, starting with young children then covering progressively older individuals in following seasons, could reduce local transmission of dengue to low levels. Simulations indicate that this strategy could avert about 7,700 uncomplicated dengue fever cases and 220 dengue hospitalizations per 100,000 people at risk over a ten-year period.

**Conclusions/Significance:**

Vaccination will have an important role in controlling dengue. According to our modeling results, children should be prioritized to receive vaccine, but adults will also need to be vaccinated if one wants to reduce community-wide dengue transmission to low levels.

## Author Summary

An estimated 40% of the world's population is at risk of infection with dengue, a mosquito-borne disease that can lead to hospitalization or death. Dengue vaccines are currently being tested in clinical trials and at least one product will likely be available within a couple of years. Before widespread deployment, one should plan how best to use limited supplies of vaccine. We developed a mathematical model of dengue transmission in semi-rural Thailand to help evaluate different vaccination strategies. Our modeling results indicate that children should be prioritized to receive vaccine to reduce dengue-related morbidity, but adults will also need to be vaccinated if one wants to eliminate local dengue transmission. Dengue is a challenging disease to study because of its four interacting serotypes, seasonality of its transmission, and pre-existing immunity in a population. Models such as this one are useful coherent framework for synthesizing these complex issues and evaluating potential public health interventions such as mass vaccination.

## Introduction

Dengue is a mosquito-borne disease, caused by a flavivirus with four serotypes, responsible for an estimated 500,000 hospitalizations and 20,000 deaths per year, mostly in the tropics [1], although these are probably conservative estimates. The toll of dengue may rise with the increasing range of its primary vectors, *Aedes aegypti* and *A. albopictus*, because of climate change and increasing urbanization in the developing world. Severe dengue cases (i.e., dengue shock syndrome (DSS) and dengue hemorrhagic fever (DHF)) occur primarily among children [2]. Although the mortality rate for dengue cases is low, even uncomplicated dengue fever causes considerable suffering and loss of productivity despite its short duration [3]–[5]. Because vector control has achieved only limited success so far in reducing the transmission of dengue [6]–[8], an effective tetravalent vaccine against all four dengue serotypes may be the only means to effectively control dengue. Such a vaccine could drive dengue rates to very low levels, as has the vaccine against yellow fever, which is also caused by flavivirus [9]. Since urban and sylvatic dengue transmission are not tightly linked [10], it is not inconceivable that dengue could be eliminated in urban areas with the targeted use of a highly efficacious vaccine.

Several dengue vaccine candidates are currently in development or in clinical trials [11]–[13]. Once vaccine becomes available, initially there will not be sufficient quantities to cover the up to 2.5 billion people at risk [1]. Vaccine will need to be introduced gradually, allowing evaluation of vaccine effectiveness and safety [14]. To reduce disease burden most efficiently with a limited supply of vaccine, it may be necessary to prioritize certain geographic regions or age groups for vaccination while taking into account the constraints of government vaccination programs and finances. However, with up to four competing dengue serotypes [15]–[17], seasonal vectors [18], [19], complex and potentially harmful immune responses to infections with heterologous serotypes [20]–[22], and the difficulty in formulating a tetravalent vaccine that protects against all four serotypes [13], [23], it is important to anticipate how the deployment of such vaccines will affect dengue virus transmission, and morbidity and hospitalizations caused by the disease [23]–[25].

Here, we investigate the potential effectiveness of different dengue vaccination strategies using a model of dengue transmission in a Thai population. The individual-level stochastic model was developed to match the epidemiology of dengue in a population in semi-rural Thailand that has experienced hyperendemic dengue transmission for many years. We modeled both single-year campaigns, in which part of the population is vaccinated well before the dengue season, and multi-year roll-outs, in which young children are vaccinated first and progressively older individuals are vaccinated in subsequent years as part of a catch-up campaign.

## Methods

### Simulation model

**Figure 1:**
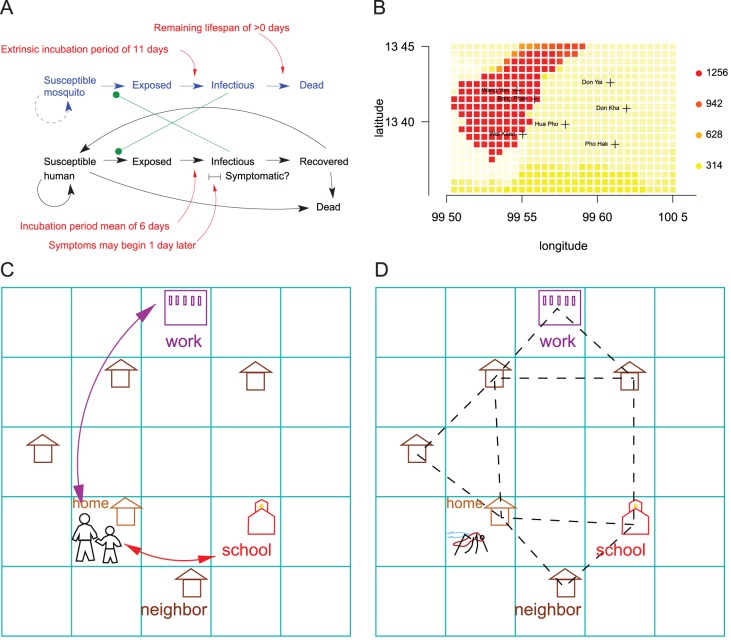
Computer simulation model of dengue transmission. (A) Natural history of dengue model. Susceptible individuals are infected by mosquitoes, and mosquitoes are infected by humans. (B) Population density of the 20 km by 30 km region surrounding Bang Phae, Thailand, at a 1 km^2^ resolution. Red indicates high population density, yellow and white for low density, as indicated in the legend in units of people per km^2^. Population density data is from GRUMP [28]. (C) Movement of humans in the model. People start and end the day at home, and go to work or school during the day. (D) Movement of mosquitoes in the model. *Aedes aegypti* are associated with a single building. Each day, they may migrate to an adjacent location (e.g., house, school, temple), as indicated by the dashed lines, with a probability of 15% and to a random location with a probability of 1%.

We developed an agent-based model of dengue transmission. The model is described in detail in Text S1. In brief, the model uses a synthetic population based on the demography of Ratchaburi, Thailand. In the model, individual humans spend time at home, work, or school, and can be susceptible, exposed, infectious, or recovered with respect to each of the four dengue serotypes. Uninfected mosquitoes, which can not transmit dengue, reside in buildings until they become infected by biting a viremic human host, at which point the mosquito may travel among nearby buildings. Exposed mosquitoes become infectious to humans after an extrinsic incubation period and remain infectious until they die (Figure 1A). Humans are immune to all serotypes for 120 days after recovering from infection. After 120 days, they are susceptible to serotypes to which they had not been exposed [26].

Secondary cases may have severe outcomes (i.e., DSS/DHF) at an age-specific proportion (Text S1). Secondary infections are otherwise treated the same as primary infections in the model except that viremia resolves one day faster [27].

We describe the synthetic population created for the model in detail in Text S1. Briefly, the model represents a 20
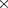
30 km area surrounding Bang Phae, Ratchaburi, Thailand (Figure 1B). We populate each square kilometer with households to match population density estimates [28]. The households are randomly drawn from the household microdata from the census of Ratchaburi province. By drawing households from census microdata, we obtain realistic age and gender distributions both within the households and in the overall population (Text S1). The synthetic population has 207,591 individuals.

Within each square kilometer, individual households, schools, and workplaces are assigned random locations. Children of the appropriate age are sent to the elementary school (ages 5 to 10 years), lower secondary school (ages 11 to 14 years), or upper secondary school (15 to 17 years). People of the appropriate age are assigned workplaces according to a gravity model in which people tend to commute to locations that are nearby and have a relatively high population density. Workplaces have an average of 20 workers, who occupy the same location during the workday.

During the morning and evening hours, people are at home, and they may go to school or work during the rest of the day (Figure 1C). Individuals symptomatically infected with dengue may stay at home until they recover. One consequence of this behavior is that there is more dengue transmission in households than at workplaces when dengue is symptomatic. Mosquitoes tend to stay in the same location (i.e., house, workplace, or classroom), but may migrate to adjacent locations with a fixed probability per day (Figure 1D and Text S1). Occasionally, the simulated infected mosquitoes will migrate to a random distant location to account for occasional long-distance travel. Because simulated mosquitoes migrate to adjacent locations with the same probability regardless of distance, they will travel farther in more sparsely inhabited regions.

To simulate multi-year epidemics, we make two simplifying assumptions: 1) there is no correlation of prior exposure to dengue within households and 2) household structures do not change over time. After simulating a single year of dengue transmission, we “age” the population by setting the immune status (both prior infections and vaccination) of all individuals of age 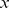 to that of randomly selected individuals of age 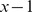 and resetting the immune status (to nave) of all people less than 1 year old. In other words, the population and households stay constant, while the immune statuses of individuals are transferred or reset each year. Thus, we account for the fact that older people will have more exposure to dengue as the simulation runs over multiple years. This approach introduces a few potential problems. One might expect changes in population structure, that could lead to the an age shift in dengue cases [29]. Therefore, to minimize the effects assuming a constant population structure, we do not run the model beyond ten years. The advantage of our approach is that the complex dynamics of household structures such as births, deaths, and marriages do not need to be included in the model. These processes are extremely difficult to simulate realistically but would be required to maintain plausible age distributions within households, schools, and the workforce. Also, the correlation of immune statuses within households and within geographic areas is disrupted in the multi-year model [30]. It also makes it impossible to trace the immune history of an individual person, since an individual's prior exposure to dengue and vaccination history will be copied from a randomly selected younger person each year. However, the population-level history of exposure to the circulating strains of dengue will be correct.

### Estimated dengue serotype-specific exposure in Thailand

In the model, individuals are assigned to have immunity from prior exposure to the four serotypes of dengue based on their age. The age-specific immune profile is based on two sources of data on the prevalence of serotypes in Thailand. Thailand's Ministry of Public Health releases an “Annual epidemiological surveillance report” that summarizes dengue serotype surveillance data. Reports from 2000–2009 are available at epid.moph.go.th, which we summarize in Table S1. For 1973–1999, we use data from a surveillance study based on children hospitalized at the Queen Sirikit National Institute of Child Health in Bangkok, as published in [31] (Table S2). Although we should be cautious about concatenating data from different sources, many of the cases reported to the Ministry of Public Health are 10–14 years old, so the populations in these two datasets are reasonably comparable.

**Figure 2:**
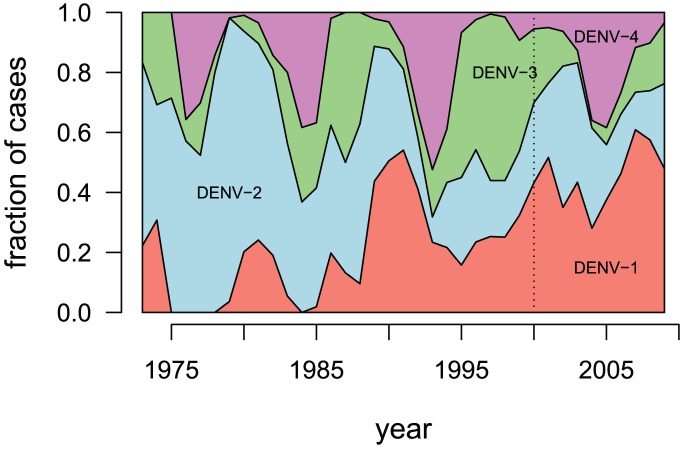
Estimated relative prevalence of the 4 serotypes in Thailand. The data are from two sources: Thailand's Health Ministry from 2000–2009 (available at http://epid.moph.go.th) and the Queen Sirikit National Institute of Child Health in Bangkok from 1973–1999 [31]. The vertical dotted line indicates the point of transition between the two datasets. Simulated prior immunity to each of the four dengue serotypes for individuals in the model were based on their ages and these data.

We estimate the age-specific immunity to the four dengue serotypes in our model. We assume that the level of exposure to dengue each year was such that 11% of nave individuals would be infected, based on studies in nearby Vietnam [32], [33]. To determine the contribution of the four serotypes to this constant annual exposure to infection, we estimate the relative prevalence of the 4 serotypes by combining the Thailand's Health Ministry's national data from 2000–2009 (available at http://epid.moph.go.th) and Queen Sirikit National Institute of Child Health in Bangkok from 1973–1999 [31] (Figure 2).

For each of the years for which we have serotype prevalence estimates, we randomly selected 11% of the population who was alive in that year (i.e., was 0 years old or older) to be exposed to dengue, and for each individual simulated exposure to a single serotype drawn from that year's prevalence data. Individuals exposed to a serotype are considered to be permanently immune. For years before 1973, we performed the same procedure, except that we assumed that the serotype prevalence was the mean serotype prevalence from 1973–2009. The mean serotype prevalences are 9.8%, 14.6%, 7.5%, and 5.2% for DENV-1, DENV-2, DENV-3, and DENV-4, respectively. In other words, we assumed that there was a constant 11% exposure to dengue (sufficient to infect) for all individuals, regardless of age or immune status, and that exposure to a serotype at any point in an individual's past grants sterilizing immunity to that serotype. In other words, each person who is exposed to dengue each year is exposed to exactly one serotype of dengue, and he or she gains sterilizing immunity to that serotype if he or she was not already immune from prior exposure.

**Figure 3:**
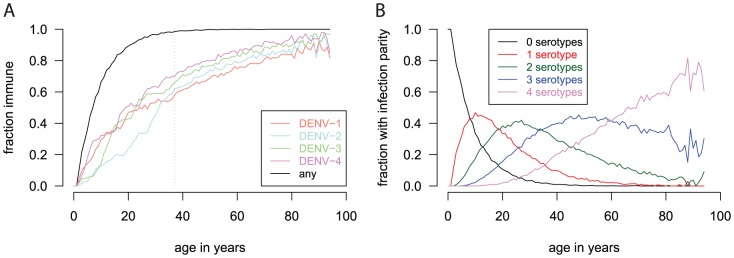
Prior exposure to the four dengue serotypes in the model's synthetic population. Simulated pre-existing immunity to the dengue serotypes in Bang Phae by age. We assume constant relative serotype prevalence before 1973, which corresponds to the vertical dotted line. Surveillance data is scaled as described in Text S2. (A) The age-specific fraction of the population immune to the four dengue serotypes. The black curve is the fraction of the population exposed to one or more of the serotypes (i.e., infection parity 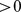). (B) The age-specific fraction of the population immune to a given number of the four dengue serotypes (infection parities). The black curve the fraction of the population exposed to none of the serotypes (i.e., infection parity 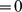).

Because the four serotypes have different symptomatic fractions, surveillance data give a skewed representation of the number of individuals infected by each serotype. We re-scaled the number of cases for each of the four serotypes in the historical data as described in Text S2. By scaling the historical surveillance data, the population-level immunity to the four serotypes changes, with increased levels of immunity to less pathogenic serotypes than if the unadjusted surveillance data were used. Figure 3 shows the age-specific immunity to dengue in the synthetic population.

## Results

### Simulating a single dengue season

**Figure 4:**
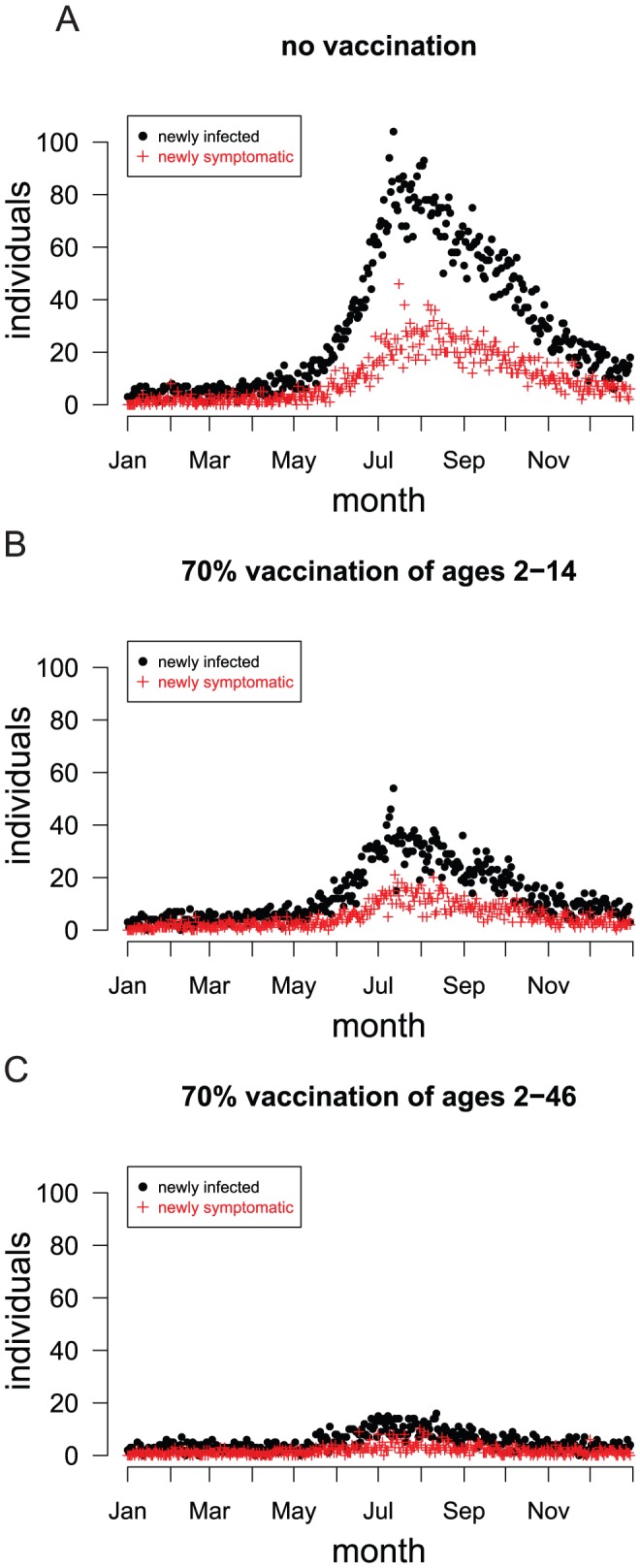
Simulated dengue incidence in a single year. Each plot shows the daily number of newly infected and symptomatic people from a single representative stochastic simulation. (A) A simulation in which no vaccination took place (baseline scenario). (B) A randomly selected 70% of the population aged 2 to 14 years was vaccinated. (C) A randomly selected 70% of the population aged 2 to 46 years was vaccinated. The vaccine confers protection to 70% of vaccinees in the model.

We simulated a single year of dengue transmission in Ratchaburi, Thailand (Figure 1B). Dengue seasonality was simulated by modeling the monthly mosquito population to conform to mosquito count data from Thailand (Text S2). To seed the epidemic, we randomly selected two people to expose to each of the four dengue serotypes for each simulation day (i.e., eight total per day, or 1.4% of the population per year). Pre-existing immunity protects many of these individuals (Figure 3), so only a few actually become infected each day. This constant seeding represents the repeated introduction of dengue from neighboring unvaccinated regions and prevents dengue from being eradicated in the model. Simulated epidemics peak in July–August (Figure 4A), about two months later than the peak in the mosquito population, which is in May–June (Text S2). This delay of dengue activity after mosquito activity is consistent with observations [34]–[36]. The lag is caused by the long mean generation time, i.e., time between when one human infects another through infected mosquitoes, of 24 days (Text S2).

**Figure 5:**
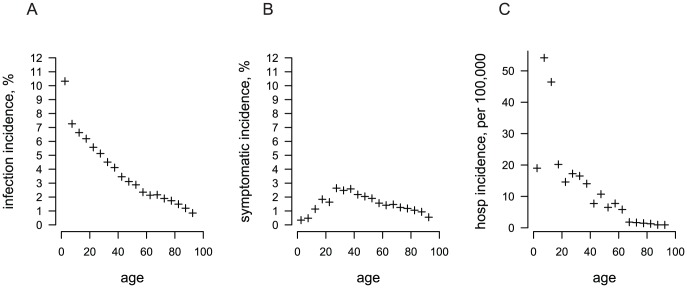
Simulated incidence of dengue infection and illness by age in a simulated season. Plotted are the average age-specific (A) infection incidence, (B) dengue fever incidence, and (C) hospitalized DSS/DHF incidence per year from fifty stochastic simulations and aggregated in 5-year age brackets.

The simulated dengue season produced a 5% infection attack rate with some stochastic variation among runs (Table 1). Because of age-specific immunity from prior exposure (Figure 3), most of the infections occur in children (Figure 5A). The 1.7% dengue illness attack rate is consistent with the estimated 2% observed in children in Ratchaburi in the 2006–2007 season [37], [38]. There were 39 severe cases requiring hospitalization per 100,000 individuals in a simulated dengue season, primarily among school-aged children (Figure 5C). The age distribution of severe cases is largely a consequence of the high inherent risk of severe outcome upon secondary infection for this age group as described in Text S1).

**Table 1:** Single year dengue simulation results.

pre-vaccination	infected per	cases per	hosp per	vaccinated	hosp averted
	100,000	100,000	100,000	per 100,000	per 100,000 vacs
0%	5,027 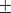 472	1,691 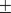 183	38.9 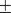 3.7	—	—
Ages 2–14, 30%	3,796 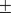 394	1,376 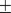 167	27.9 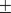 2.9	6,673	165
50%	3,191 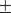 262	1,198 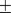 113	22.6 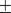 1.9	11,121	147
70%	2,615 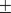 170	996 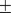 88	17.9 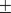 1.2	15,570	135
Ages 2–46, 30%	2,353 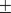 209	816 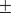 83	17.6 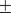 1.6	22,303	96
50%	1,434 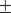 110	493 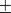 47	10.5 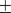 0.9	37,172	76
70%	904 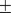 65	316 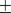 33	6.5 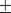 0.5	52,040	62

Various fractions of the population (0%, 30%, 50%, and 70%) from 2 to 14 years old or from 2 to 46 years old were pre-vaccinated and fifty simulations were run per scenario. The vaccine protects 70% of vaccinees against infection. The averages and standard deviations of the numbers of infections, symptomatic infections (cases of uncomplicated dengue fever), severe cases requiring hospitalization, and vaccinations per 100,000 individuals from fifty stochastic simulations per scenario are reported. Also reported are the average numbers of hospitalizations averted per 100,000 vaccinations.

We report the total number of uncomplicated and severe (DSS/DHF) cases produced by our model assuming perfect surveillance. Estimates of reporting rates would be needed to compare our modeling results with actual surveillance data. Wichmann et al. estimated that, among children, total dengue cases in Thailand may be underreported by a factor of 8.7 and severe (inpatient) dengue cases by 2.6, with less underreporting in school-aged children than in younger children [37]. Underreporting among adults is likely higher [39] but is difficult to quantify due to the lack of prospective cohort or active surveillance studies that include adults [25], [40]. The age distribution of symptomatic cases produced by our model is older than we had anticipated (Figure 5B). This discrepancy may be due to underreporting of adult dengue cases by routine surveillance, which would skew the age distribution downward. It is also possible that the model overestimates cases among older individuals. Antibodies from exposure to multiple serotypes may be cross-protective, so third and fourth dengue infections may be rare or only mildly symptomatic [41]. The model is sensitive to changes in the maximum permissible infection parity (Text S3). Reducing the maximum infection parity to two or three not only greatly reduces the attack rate, but also shifts the age distribution of cases downward.

During the simulated seasonal peak of dengue transmission, a single person infected an average of 1.9 to 2.3 others, depending on the serotype (Text S2). This is the reproductive number, 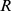, a measure of transmissibility that takes the background of immunity from prior exposure into account. A rough estimate of the critical vaccination fraction to stop transmission in the population, assuming a randomly mixing population, is 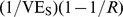, where 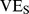 is the vaccine efficacy against infection [14]. For example, a vaccine with 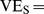
70% for all four serotypes would have a critical vaccination fraction of about 80%, while a vaccine with 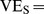
90% would have a fraction of 60%. Although these figures give a crude starting point for thinking about what level of vaccine coverage may be needed to eliminate dengue in a population, more detailed calculations are needed, as described below.

### Simulating vaccination before a single dengue season

We simulated vaccinating the population to protect them before a single dengue season. Recently, an observer-blind, randomized, controlled, phase 2b vaccine trial was conducted with a tetravalent dengue vaccine [38]. The serotype-specific estimated vaccine efficacy for confirmed dengue illness ranged from 55–90% for serotypes 1, 3 and 4, but was close to 0% for serotype 2. Partially based on this, we investigate the 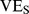 with a point estimate of 70% for all four serotypes, and we assumed that vaccine-derived immunity does not wane. We do sensitivity analyses with the 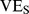 ranging for 50–90% and with the 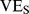 set to zero for a single serotype. This vaccine candidate has been tested in 1–45 year-olds and requires three courses administered over the course of one year [12]. If one conservatively assumes that an individual is only protected after receiving all three doses, then only those 2 years and older could be protected by vaccine. Therefore, in the simulation results presented below, we simulated the vaccination of individuals between 2 and 46 years old.

Vaccinating 70% of children 2 to 14 years old would reduce the number of dengue infections by 48%, uncomplicated dengue fever cases by 41%, and severe dengue cases (DSS/DHF) by 54% in a single year (Table 1 and Figure 4B). The proportion of uncomplicated cases prevented is lower than the proportion of infections because infected children are less likely to become symptomatic with dengue fever than adults (Text S1), but the proportion of severe cases prevented is higher than the proportion of infections because children are more likely to develop DSS or DHF upon secondary infection than adults (Figure 5 and Text S1). Because children from ages 2 to 14 years comprise only 22.2% of the population, vaccinating them does not reach the estimated 80% coverage required to control dengue. Extending the vaccination to include adults up to 46 years old reduced the number of infections by 82%, dengue fever cases by 81%, and severe cases by 83% (Table 1). Vaccinating 70% of individuals aged 2 to 46 years would result in 52% coverage of the total population. Thus, vaccinating 70% of this population greatly reduces the seasonal peak (Figure 4C), while vaccinating a smaller fraction of this population is less effective. Simulations in which the vaccine had higher efficacy produced better, but similar, results (Text S4). However, a vaccine that protects against only three of the four serotypes is substantially less effective than one that offers good protection against all four (Text S4). Because the four serotypes compete in our model, reduction in the circulation of three of the serotypes could result in *increased* transmission of the remaining serotype, at least in the short term.

Those who are *not* vaccinated receive indirect protection when enough of the remaining population is vaccinated. In our simulations, those who are over 46 years old are never vaccinated, but people in this age group were 44%, 61%, and 71% less likely to become infected when 30%, 50%, and 70% of those from ages 2 to 46 years were vaccinated. Unvaccinated individuals from ages 2 to 46 were 60%, 80%, and 91% less likely to become infected when 30%, 50%, and 70% of this age cohort were vaccinated.

**Figure 6:**
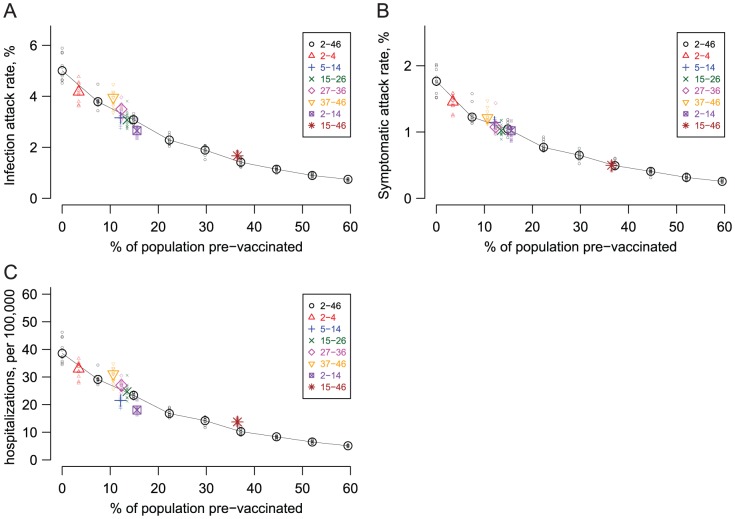
The simulated effects of pre-vaccinating different age cohorts against dengue. The larger points represent the median attack rates (y-axis) of ten stochastic simulations run when a percentage of the total population (x-axis) is pre-vaccinated. The results from the individual simulations are plotted as small points to show the stochastic variation. The black Os, connected by lines, represent the effect of pre-vaccinating different fractions of individuals from 2 to 46 years old, from 0 to 80% of this cohort, which translates to 0 to 59.5% of the total population. The other symbols are the results from targeting 70% of individuals in narrower age cohorts (expressed in years in the legends) for pre-vaccination. Pre-vaccinating a particular age group can be considered more efficient than vaccinating an equivalent number of people from ages 2 to 46 if it results in a lower attack rate (i.e., the point falls below the line). The vaccine confers immunity to 70% of those vaccinated in the model. (A) Overall infection attack rate vs. pre-vaccination fraction. (B) Overall symptomatic (uncomplicated dengue fever) attack rate vs. pre-vaccination fraction. (C) Overall DSS/DHF cases (hospitalizations) vs. pre-vaccination fraction.

Certain age groups could be prioritized to receive vaccine. Younger people have the least prior exposure, so they would be the most likely to become infected with and transmit dengue. Simulations demonstrated that vaccinating children (2–14 years old) would reduce dengue infections in the total population more than using the same number of doses to cover both children and adults (2–46 years old) (Figure 6A). However, dengue is more likely to be symptomatic in older individuals than younger (Text S1). Thus, the advantage of concentrating vaccine in children was less pronounced when observing symptomatic dengue (Figure 6B). Children are more likely than adults to have severe outcomes (DSS/DHF) upon secondary dengue infection (Text S1), and vaccinating children was more effective in reducing severe cases than vaccinating adults (Figure 6C). For example, vaccinating 70% of children from ages 2 to 14 years would reduce the overall severe case rate to 18.0 per 100,000, compared to 22.8 per 100,000 if the same number of individuals from ages 2 to 46 years were vaccinated. Vaccinating 70% of those from ages 15 to 46 years would reduce the overall severe case rate to 13.7 per 100,000, compared to 10.6 per 100,000 if the same number of individuals from ages 2 to 46 were vaccinated. In other words, concentrating vaccine among children should reduce hospitalizations more than vaccinating both children and adults.

### Simulating multi-year vaccine roll-outs

**Figure 7:**
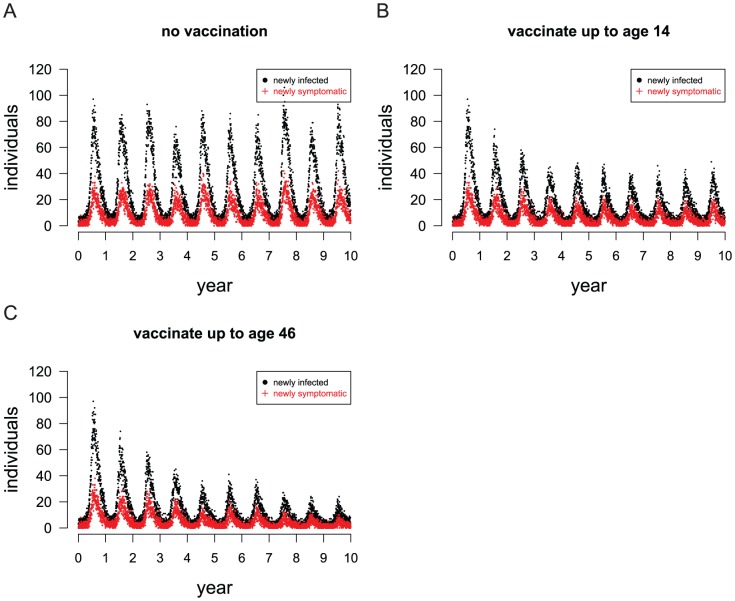
The simulated effect of vaccination on daily infection and illness incidence over ten years. (A) No vaccination. (B) Vaccine roll-out that covers only children ages 2 to 14 years. (C) Vaccine roll-out that covers children and adults ages 2 to 46 years. 70% of each age cohort is vaccinated, and the vaccine confers all-or-none protection to 70% of vaccinees. The points indicate the number of newly infected or symptomatic people during a single day in a single representative stochastic simulation.

Due to limited vaccine availability and the logistics of mass vaccination programs, dengue vaccine will probably be deployed in multi-year vaccine roll-out campaigns [42]. We simulated a vaccine roll-out that covers only children, reaching 70% of children from ages 2 to 14 years within three years, after which point only 2-year-olds are vaccinated. Specifically, we simulated the vaccination of 70% of 2 to 4 year olds in the first year, 2 year olds and 6 to 9 year olds in the second year, 2 year olds and 11 to 14 year olds in the third year, then only 2-year-olds for the following six years, as shown in Figure S1A. The incidence of dengue infections drops sharply for the first three years, after which incidence declines slowly (Figure 7B).

We also simulated a vaccine roll-out that extended the catch-up to include adults up to age 46. This roll-out targets the same age groups for the first three years as the previously described roll-out, but after this point both 2-year-olds and the youngest four unvaccinated age cohorts are vaccinated, as shown in Figure S1B. Including young adults in the catch-up caused the incidence of dengue to continue dropping rapidly after children were covered by the third year (Figure 7C and Table 2). For the roll-out that includes adults, 7,699 uncomplicated cases and 217 severe cases per 100,000 people at risk would be averted by vaccination over a ten-year period.

**Table 2:** Multi-year dengue simulation results.

	Baseline		Roll-out ages 2–14		Roll-out ages 2–46	
year	cum cases per	cum hosp per	cum cases averted	cum hosp averted	cum cases averted	cum hosp averted
	100,000	100,000	per 100,000	per 100,000	per 100,000	per 100,000
1	1,706 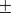 188	40 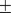 4	—	—	—	—
2	3,424 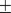 217	80 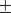 5	225	6	225	6
3	5,128 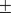 217	119 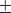 5	689	22	689	22
4	6,837 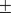 289	159 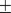 5	1,367	43	1,367	43
5	8,516 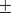 217	198 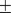 5	2,061	65	2,186	68
6	10,179 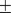 243	237 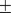 6	2,717	87	3,098	94
7	11,824 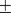 291	276 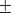 7	3,329	109	4,106	122
8	13,469 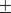 296	315 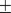 7	3,992	131	5,212	152
9	15,143 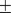 312	355 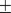 7	4,700	155	6,426	185
10	16,809 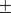 353	394 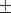 8	5,418	179	7,699	217

The yearly number of cases and hospitalizations per 100,000 individuals is estimated in 10-year simulations of dengue transmission when there is no vaccination (baseline), roll-out that covers only individuals ages 2 to 14 years, and roll-out that covers individuals ages 2 to 46 years. The vaccine protects 70% of vaccinees against infection. The cumulative uncomplicated dengue fever cases and severe cases requiring hospitalization averted are relative to the baseline scenario. Results are the averages and standard deviations from fifty stochastic simulations per scenario.

## Discussion

We used a dengue simulation model to estimate that vaccination of 50% of the population of rural Thailand could be sufficient to reduce local dengue transmission to low levels. Based on our modeling study, we conclude that at least 70% efficacy against infection for all serotypes is desirable if one wants to control dengue in a hyperendemic area, and a higher efficacy vaccine would require less careful targeting of vaccine to reduce community-wide transmission of dengue. We further showed that vaccinating children is the most efficient use of vaccine to reduce cases and hospitalizations, but control of dengue transmission would also require vaccinating adults. In addition, both vaccinated and unvaccinated people would receive protection from mass vaccination because of the considerable indirect effects of dengue vaccination. A vaccine that only protects against only three serotypes could lead to a significant reduction in overall vaccine effectiveness. Further work will be needed in order to understand how to use vaccines that may not protect against all four serotypes. Using a detailed model of dengue transmission allows one to explore strategies that target vaccines most efficiently.

To capture the complex interactions required to evaluate the effectiveness of mass vaccination with tetravalent dengue vaccines, the model includes vector population seasonality [34], [43], human mobility [44], [45], population heterogeneities, and individual vectors [19]. Thus, we have a coherent framework for modeling both dengue transmission and the effects of vaccination in a complex population. The model by necessity includes a number of assumptions and simplifications, such as the model structure, parameterization, and vaccine efficacy. The model may be sensitive to assumptions we made regarding unresolved questions about dengue immunology, such as the susceptibility of individuals after sequential infection by more than two serotypes (Text S3). Although our model qualitatively captures the epidemic dynamics of a single season of dengue in semi-rural Thailand, there are complex multi-year dynamics that we can only approximate. More realistic modeling of the prevalence cycles of the four dengue serotypes would require more complex and calibrated inter-serotype interactions (e.g., [15], [46]), and further studies are needed to quantify these effects. Furthermore, our results apply to dengue transmission in a hyperendemic area, which has a high incidence of dengue and multiple circulating serotypes. In regions with lower transmission, the levels of population immunity to the various serotypes and the force of infection would be lower, resulting in different effectiveness of mass vaccination. Models that require a great deal of regional data such as ours may need to be adapted to the specific regions of interest to produce useful results. However, our model agrees with previous model-based estimates that 50–85% of a population need to be vaccinated to reduce transmission to negligible levels [47]. Therefore, our model produces results qualitatively similar to those from simpler models that assume homogeneous mixing of the human population.

An estimated 40% of the world's population is at risk of dengue infection [48], and vaccinating this population is not feasible in the short term. The greatest need for dengue control is in areas where dengue disease is hyperendemic, primarily South-east Asia, Latin America, and the Caribbean and Pacific Islands. A coalition of non-governmental organizations, national health ministries, and vaccine manufacturers could establish priorities for allocating vaccine in publicly funded mass vaccination campaigns. Private demand might be sufficient to cover enough of the remaining population to reduce dengue transmission to manageable levels [49].

Large-scale vaccination campaigns would be both challenging and costly but could be more cost-effective than relying solely on vector control and other non-pharmaceutical interventions [50], [51]. Vaccination would not only reduce local disease burden, but may reduce the rate of evolution of dengue viral genetic changes. Education campaigns and aggressive vector control measures could complement vaccination if it is not feasible to vaccinate enough individuals to eliminate local dengue transmission. However, such non-pharmaceutical strategies are difficult to sustain, and there have been doubts about their effectiveness [6]–[8], [52]. Novel vector control strategies that involve releasing parasitic bacteria or genetically engineered mosquitoes are promising [53]–[56], but their deployment may be controversial. Given the difficulty of controlling dengue with currently available technologies, we believe that vaccination will become an essential component of dengue reduction efforts.

## Supporting Information

Figure S1 **Diagram of how vaccine roll-out occurs in the model.** In the model, we target a set of age cohorts each year for vaccination. The numbers in the diagrams indicate the ages of the population cohorts in years. The numbers in black in each row indicate the cohorts targeted each year, while the numbers in red indicate those already protected by vaccine from previous years. The set of individuals who are protected advance in age each year, so the youngest eligible cohort (2-year-olds) needs to be vaccinated every year. (A) A roll-out that covers only children from ages 2 to 14 years. (B) A roll-out that covers both children and adults from ages 2 to 46 years.
(EPS)

Table S1 **Serotype surveillance data from Thailand's Ministry of Public Health.** Data from http://epid.moph.go.th/Annual/.
(PDF)

Table S2 **Serotype surveillance data from Queen Sirikit National Institute of Child Health in Bangkok.** Data from Nisalak et al (2003).
(PDF)

Text S1 **Dengue transmission model description.**
(PDF)

Text S2 **Parameter selection for the model.**
(PDF)

Text S3 **Sensitivity of attack rates to maximum infection parity assumptions.**
(PDF)

Text S4 **Sensitivity of attack rates to vaccine efficacy.**
(PDF)
