# Supplementary material for: Controlling Dengue with Vaccines in Thailand
Source: PLoS Negl Trop Dis. 2012 Oct 25;6(10):e1876. doi: 10.1371/journal.pntd.0001876 (PMC3493390; doi:10.1371/journal.pntd.0001876)
Supplement: Table S2 — Serotype surveillance data from Queen Sirikit National Institute of Child Health in Bangkok. Data from Nisalak et al (2003). (PDF) [file pntd.0001876.s003.pdf]

Table S2. Serotype surveillance data from Queen Sirikit National Institute of Child Health in Bangkok. Data from Nisalak et al (2003).

year	year (Thai)	DENV-1	DENV-2	DENV-3	DENV-4
2000	2543	43.2%	26.7%	24.6%	5.5%
2001	2544	51.7%	24.8%	18.5%	5.05%
2002	2545	35.0%	47.1%	11.6%	6.3%
2003	2546	43.4%	39.8%	4.1%	12.7%
2004	2547	28.1%	33.4%	2.5%	36.0%
2005	2548	37.5%	18.4%	5.7%	38.4%
2006	2549	46.3%	19.8%	7.2%	26.7%
2007	2550	60.9%	12.5%	14.9%	11.7%
2008	2551	57.5%	16.4%	15.9%	10.2%
2009	2552	47.6%	28.2%	20.2%	3.4%
