# Supplementary material for: Controlling Dengue with Vaccines in Thailand
Source: PLoS Negl Trop Dis. 2012 Oct 25;6(10):e1876. doi: 10.1371/journal.pntd.0001876 (PMC3493390; doi:10.1371/journal.pntd.0001876)
Supplement: Text S1 — Dengue transmission model description. (PDF) [file pntd.0001876.s004.pdf]

Online Supporting Text S1: Dengue transmission model description

Dennis L. Chao[1], Scott B. Halstead[2], M. Elizabeth Halloran[1,3], Ira M. Longini, Jr[4,*]
1 Center for Statistics and Quantitative Infectious Diseases, Vaccine and Infectious Disease Division, Fred Hutchinson Cancer Research Center, Seattle, Washington, USA
2 Dengue Vaccine Initiative, Seoul, South Korea
3 Department of Biostatistics, School of Public Health, University of Washington, Seattle, Washington, USA
4 Department of Biostatistics, College of Public Health and Health Professions, and Emerging Pathogens Institute, University of Florida, Gainesville, Florida, USA
∗ E-mail: Corresponding ilongini@ufl.edu

S1 Dengue transmission model description

We developed a model of dengue transmission that represents individual infected mosquitoes (Section S1.1) and human hosts (Section S1.2). Section S1.3 describes how dengue is transmitted between humans and mosquitoes and vice versa. The model runs in discrete time in one-day time steps. Typically, the model is run for a single year, but it can be run for multiple years as described in the Materials and Methods section in the main text. The model was written in C++ (GNU Compiler Collection version 4.6.3 for 64-bit x86) and uses the GNU Scientific Library for random number generation (GSL-1.15, available at http://www.gnu.org/software/gsl/). We used the R statistical computing software version 2.14.1 for data analysis and plotting [1].

S1.1 Mosquito model

The lifespan of a mosquito affects its ability to acquire and transmit dengue. We assume that mosquitoes have a lifespan determined by a hazard function [6]. The hazard function is logistic: $H(t) = \frac{ae^{bt}}{1+\frac{as}{b}(e^{bt}-1)}$,

Table S1.1. Model parameters.

Parameter	value	source
mean incubation period	6.0 days	[2]
symptom onset	0–2 days	[2, 3]
viremic period	4–5 days	[2]
symptomatic fraction	varies by age	[4]
symptomatic infectiousness	unknown	—
days of complete cross-immunity after recovery	120 days	—
extrinsic incubation period	11 days	[2]
mean mosquito lifespan	18 day mean	Section S1.1
B_t, biting time distribution	0.08,0.76,0.16	[5]
daily probability of mosquito migration	0.15	Section S2.1
β_{PM}	0.1	person-to-mosquito transmission (Section S2.1)
β_{MP}	0.25	mosquito-to-person transmission (Section S2.1)
$\mathsf{VE_S}$	0.5–0.9	—
$\mathsf{VE_P}$	0, 0.5	—
$\mathsf{VE_I}$	0, 0.5	—

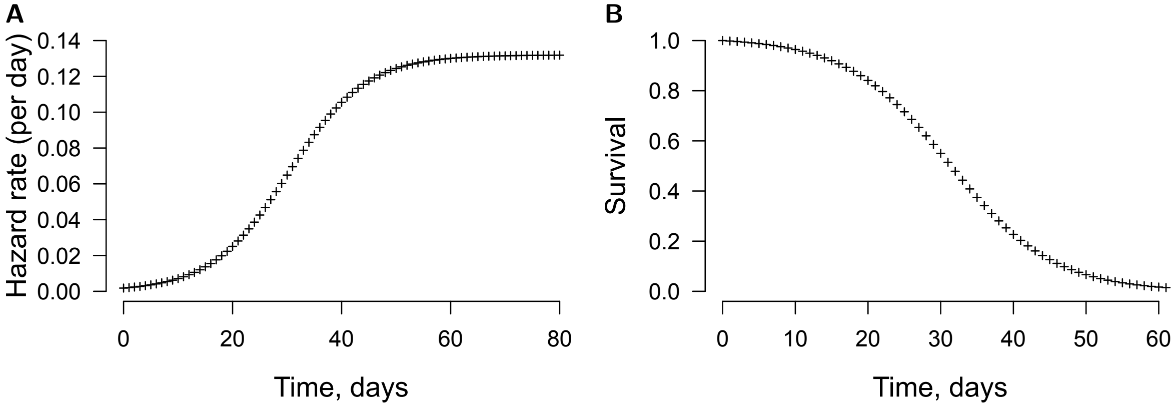

Figure S1.1. Simulated mosquito lifespan. (A) Female *Aedes aegypti* mortality, from [6]. (B) A survival curve derived from the hazard.

where $a = 0.0018$, $b = 0.1416$, $s = 1.0730$ for female *Aedes aegypti* (Figure S1.1). These estimates are for caged mosquitoes, and mosquitoes in the wild may have shorter lifespans due to predation. The model's dengue dynamics were calibrated using the caged mosquito estimates. We created a survival function based on the discretized hazard. By assuming the maximum age is 60 days, we compute the steady state age distribution of living (female) mosquitoes.

We assume that each building in the model has the capacity to maintain a certain number of mosquitoes, which could correlate with the number of breeding sites. The number of mosquitoes (per building) fluctuates according to a seasonality function that can change weekly (Section S2.4). In the absence of viremic humans, the mosquitoes remain susceptible and are represented as a population per building rather than as individual, mobile mosquitoes.

We define the vector B_t to be how much mosquitoes prefer to bite at different times of day, where t is a portion of a single day. We use the female biting behavior in the rainy season in Thailand to estimate B_t: 8% of biting takes place in the first two hours of the day (before 9am), 76% of biting takes place between 9am and 5pm, 13% takes place between 5pm and sunset, and 3% of biting takes place after sunset [5]. We assume that this distribution reflects the mosquito's feeding time preferences rather than the availability of hosts.

Infected mosquitoes are represented as mobile, individual agents that are generated in buildings with infected humans. For each building, we calculate f_v, the fraction of people who are viremic weighted by B_t, based on when each individual is present, to account for mosquito biting time preferences (Table S1.1). We draw from the binomial distribution $\mathcal{B}(n, \beta_{PM} f_v)$ to determine the number of mosquitoes to be infected at that location in a day. β_{PM} is the daily probability that a susceptible mosquito bites a human multiplied by infectiousness. The infecting serotype is drawn from the distribution of serotype frequencies at that location, also weighted by B_t. Thus, individuals who are in the building when the mosquitoes prefer to bite are most likely to infect the mosquitoes. Also, if there are more uninfected individuals, it is less likely that a mosquito will become infected. The age of the newly infected mosquito is drawn from the pre-calculated age distribution of uninfected mosquitoes, A, as described above. We also draw the mosquito's age of death by drawing from the age distribution of mosquitoes older than itself. The building where the mosquito was infected has one less susceptible mosquito until the newly infected mosquito's death. The newly infected mosquito enters the "exposed" state for an extrinsic incubation period of exactly 11 days until becoming infectious [2] (Figure 1A). After the extrinsic incubation period, exposed mosquitoes become infectious, and can infect susceptible humans in the model. Infectious mosquitoes potentially

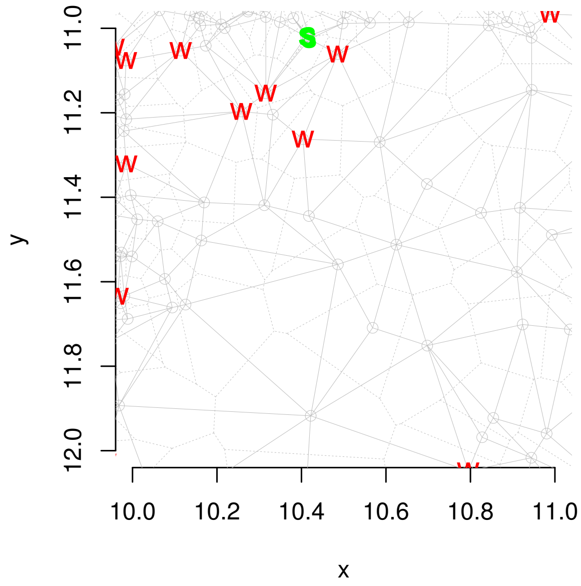

Figure S1.2. Locations in the Bang Phae model are linked by a Delaunay triangulation. In this plot, which covers 1km^2, workplaces are indicated by red W's, schools by green S's, and homes by gray circles. The solid gray lines connect neighboring locations, defined by the triangulation, and are the routes along which mosquitoes travel. The schools are modeled as tight clusters of classroom locations, and the green S in the plot is actually six adjacent primary school classrooms, each associated with about 30 children and one adult. The Voronoi diagram is shown as gray dotted lines. Each location is surrounded by a Voronoi "cell" defined by these lines, and each cell can be considered to be the territory of mosquitoes occupying the location.

transmit dengue with the probability of β_{MP} per day. This parameter is conceptually the product of a daily biting frequency and the mosquito-to-human transmissibility. Thus, β_{MP} is the daily probability of transmission per infectious mosquito in a fully susceptible human population. In the model, each mosquito delivers an "infectious" bite with the probability of β_{MP} each day, at which point a randomly selected individual at the mosquito's location, weighted by the amount of time each individual spends at that location, is infected if and only if that person is susceptible.

Infected mosquitoes are mobile in the model. In the model, each infected mosquito is associated with a single building, which may be a household, workplace, or school, and they may migrate to adjacent buildings with a probability of 0.15 per day (Section S2.1) and to a randomly chosen building with a probability of 0.01 to account for sporadic long-range travel in a manner similar to the model in [7] (Figure 1C). Adjacency among buildings is determined using a Delaunay triangulation implemented in R's deldir package [1]. There are a total of 60,126 locations. As a consequence of traveling along this network, mosquitoes will only travel short distances in densely populated areas and longer distance in sparser areas, which was observed in [8]. See Figure S1.2 for an illustration of the triangulation.

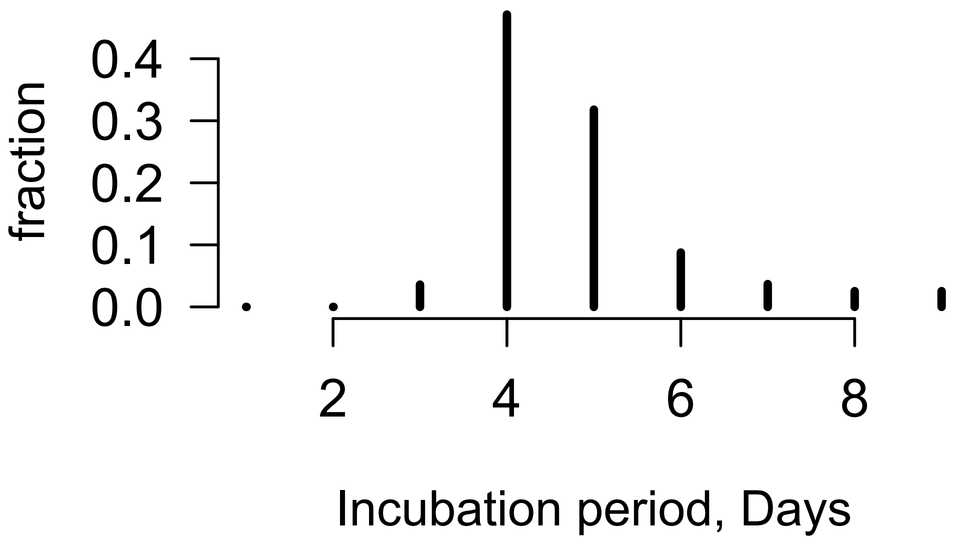

Figure S1.3. Distribution of intrinsic incubation periods. Data from [2].

S1.2 Human model

Individual humans have a fixed daily pattern of movement in the model. Because we are interested in dengue transmission, we only model the daytime behavior of humans, when *A. aegypti* are biting. We divide the day into three portions: morning (before 9am), the work day (between 9am and 5pm), and early evening (after 5pm). People will be assigned locations (e.g., home, work, school, temple) for each of these time slots. We assume that people spend their mornings and early evenings at home and go to work (if "employed") or school during the day. Symptomatic individuals might stay at home during the day, as described in Section S1.4. Based on http://web.nso.go.th/en/survey/timeuse/time_use.htm, people in Thailand are generally at home eating between 7-8AM and 6-7PM. This gives us a good idea of when people are at home, assuming they take their morning and evening meals at home.

S1.3 Dengue natural history and the effect of vaccine

Susceptible people in the model are infected when they are bitten by an infected mosquito, as described in Section S1.1. The probability that an individual is bitten is determined by the proportion of time they spend in buildings with infected mosquitoes. Once a human is infected, we draw incubation periods from an empirical distribution (Figure S1.3) derived from observations of DENV-1 challenge studies from the US Army in the Philippines [2].

We assume that symptoms begin 1 day after infectiousness, which was observed in a study of DENV-4 [2]. Infectiousness lasts 5 days, which is consistent with [2] in which infectiousness is high from 1 day before to 3 or 4 days after fever onset. We assume that infectious individuals have a constant level of infectiousness, regardless of serotype or prior heterologous exposure. Secondary infections (i.e., infections after exposure to one or more serotypes) are assumed to have the same rates of causing dengue fever as primary infections (but higher rates of severe cases, see Section S1.4), and viremia during secondary infections resolves one day faster than during primary infections in the model (3 days after symptom onset instead of 4) [9].

Symptomatic individuals may choose to stay home for the remaining duration of illness with a 50% probability per day, starting with the first day of symptoms. See Section S1.4 for a description of who

becomes symptomatically infected in the model. Hospitalization is not represented in the model, and the number of hospitalizations can be estimated by taking a fraction of the primary and secondary symptomatic individuals at the end of a simulation run.

Vaccine-induced protection is modeled as having three components as described in [10]. $\mathsf{VE_S}$ is the probability that a vaccine will confer immunity against infection, $\mathsf{VE_P}$ is the reduction in the probability of becoming symptomatic given infection, and $\mathsf{VE_I}$ is the reduction in infectiousness. The simulated vaccine provides the same level of protection against all 4 serotypes. In the model, vaccine confers all-or-none protection, so that a fraction of those vaccinated (i.e., $\mathsf{VE_S}$) can not be infected by any serotype. In Section S4, we relax this assumption in a sensitivity analysis.

We assume that naturally acquired infection also grants immunity. In the model, infected individuals acquire sterilizing immunity to the infecting serotype and complete but brief (120 days) cross-immunity to other serotypes after recovery from infection

S1.4 Pathogenicity of dengue

During primary infection with DENV-1, the risk of symptomatic dengue (uncomplicated dengue fever) increases with age [4]. We use the data from [4] to compute pathogenicity for DENV-1 and DENV-3 infections in individuals in the model, with adults having a 100% symptomatic fraction (Figure S1.4). We assume that secondary infections have the same probability of producing a case of dengue fever as primary infections.

Primary infection with serotypes 2 and 4 are mostly inapparent, while infection with 1 and 3 are more often symptomatic [11,12]. The DENV-2 strain circulating in Cuba in 1997 appeared to be asymptomatic in naïve individuals [13]. In the model, we assume that DENV-2 and DENV-4 are less pathogenic than DENV-1 and DENV-3 and use a rescaled version of the age-specific pathogenicity curve with pathogenicity peaking at 30% in naïve adults rather than 100%.

After a simulation is run, we compute the number of dengue-related hospitalizations based on the number of infections. We assume that hospitalizations are due to secondary (heterologous) infections. Therefore, we count the number of simulated heterologous infections, then use age-specific estimates of the number of DHF/DSS-related hospitalizations per secondary infection from [14], as shown in Figure S1.5.

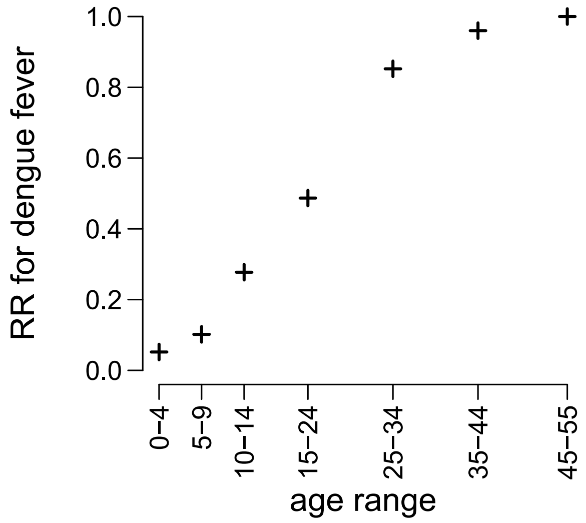

Figure S1.4. Relative risk by age of developing dengue fever (becoming symptomatic) after infection by DENV-1 in a naïve population. Data from [4].

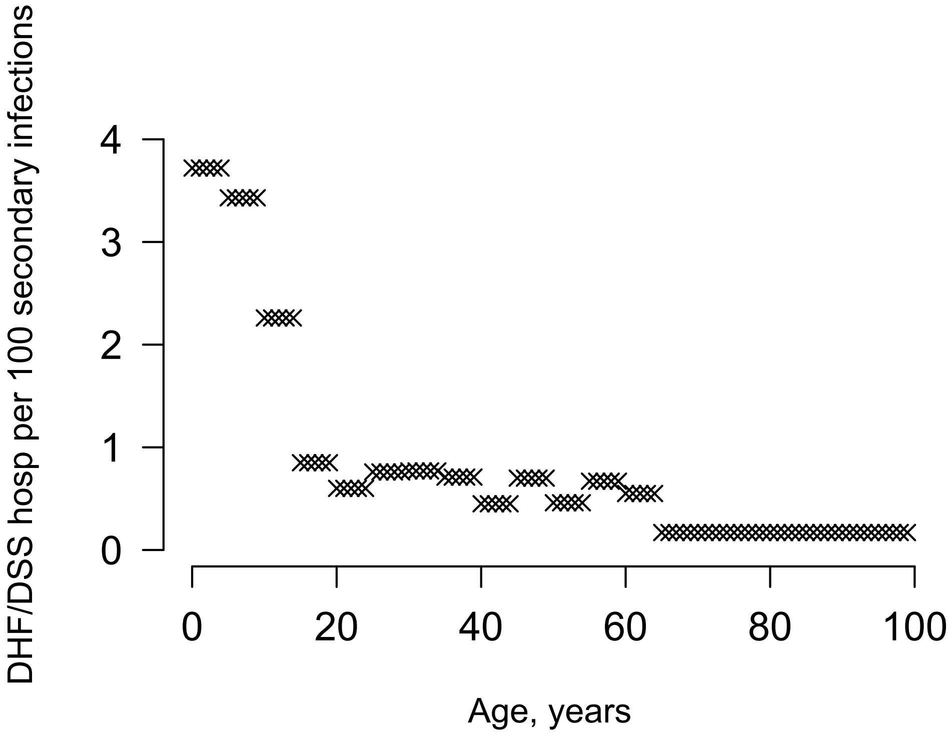

Figure S1.5. Risk of DSS/DHF hospitalization per 100 secondary infections. Data from [14]. Estimates for ages less than 3 are assumed to be the same as the estimate for age 3. Estimates for ages greater than 65 are assumed to be the same as that for age 65.

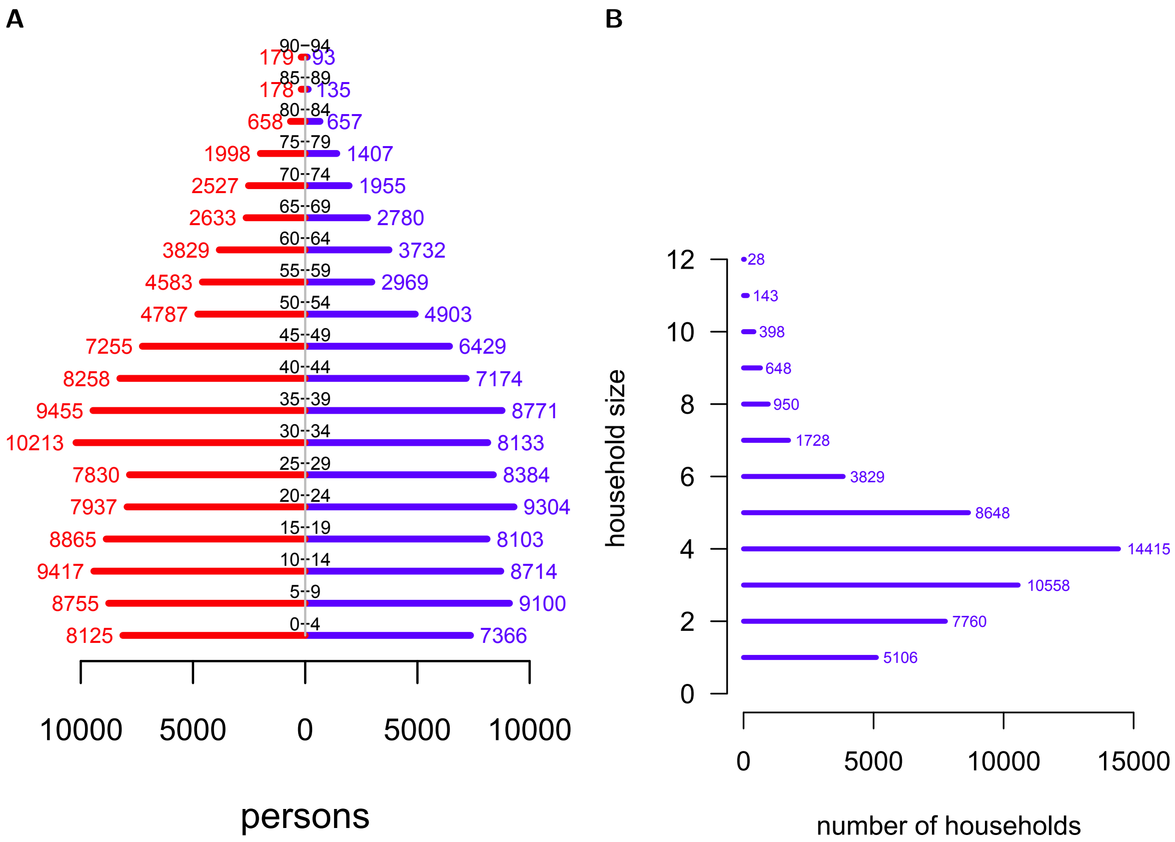

Figure S1.6. Synthetic population demographics. (A) The distribution of ages by gender in the synthetic population based on Ratchaburi. Males are in blue, females in red. (B) The distribution of household sizes.

S1.5 Synthetic population for Bang Phae, Ratchaburi, Thailand

The Bang Phae district in Ratchaburi is semi-rural, and consists of 7 sub-districts (Bang Phae, Don Kha, Don Yai, Hua Pho, Pho Hak, Wang Yen, and Wat Kaeo), 65 villages, and 49,506 residents (11,472 households). To make the model specific to the site, we use population density, family structure, mobility data, mosquito population seasonality, and cultural factors.

We create a synthetic population for a rectangular area around the district, from 13°35′–13°45′N and 99°50′ – 100°5′E, which is 20×30 km. We use population density estimates from GRUMP [15], which has estimates at a 1km^2 resolution (Figure 1B). In the synthetic population, we randomly place households within each 1km^2 grid cell to match the GRUMP data. Households are drawn from the Integrated Public Use Microdata Series, International, (IPUMSI) database [16]. These data contain the ages and genders of members of 2040 households sampled in Ratchaburi from the 2000 Census. The synthetic population has 207,591 individuals (Figure S1.6), which is close to the 207,437 residents according to GRUMP.

We model the school structure in the manner described in [17] for rural Thailand. All children from 5 to 10 years old go to elementary school. In our synthetic population, this is 21,259 children. We create 182 elementary schools in order to have an average school size of 117 students. We place 50% of elementary schools within 100 meters (using an L_1 norm) of randomly selected homes, so that there are more schools

where there are more people, and the remaining 50% randomly across the whole area. We assume that 83% of the 14,727 children from 11 to 14 go to one of the 129 lower secondary schools (average size 95). We assume 58% of the 9,855 children from 15 to 17 go to one of the 83 upper secondary schools (average size 69). We place half of these schools near homes and half randomly, as we do for elementary schools. We assign the stated fractions of children of the appropriate ages to the nearest school.

According to census data, classrooms have about 20-35 students (http://web.nso.go.th/topten/305.htm and http://web.nso.go.th/topten/303.htm). All schools that are large enough are split into classrooms of about 30 students. More specifically, we set the number of classrooms in a school by dividing the number of students by 30 and rounding and also requiring at least one classroom per school. If a school requires more than one classroom, the appropriate number of classrooms are created within a 10x10 area around the original school, and students are randomly divided among the classrooms. One adult is assigned per classroom, as described below.

We assign activities for the adults and the children who don't go to school using the procedure described below:

1. The oldest female (who is 15 or older) in each household with children 5 years old and younger stays home during the day (i.e., their workplace is their home). There are 19,352 children 5 and under in 15,799 households. Most of these households (15,554) have a qualified female, who stays home with the child. The remaining children 5 and under (in the remaining 245 households) are unsupervised at home.

2. For each classroom, one adult between 21 and 64 who live within 2km is randomly selected to work there.

3. Of the remaining individuals from 12–64 years old, some fraction (70%) will be assigned to work outside the home. We use a gravity model to determine how far employed individuals travel to go to work [18, 19]. To determine the distribution of destinations for workers in a given community, we use the following formula from [19]:

$$C_{ij} = \theta \frac{P_i^{\tau_1} P_j^{\tau_2}}{d_{ij}^{\rho}} \tag{1}$$

   where C_{ij} is the workflow from cell i to j, d_{ij} is the distance between i and j, P_i is the population of community i, θ is a proportionality constant, and τ is used to tune the dispersal. Each cell is a 1km^2 square. Based on [19], we set $\tau_1 = 0.30$ and $\tau_2 = 0.64$. To reflect the lower mobility of semi-rural Thailand, we set $\rho = 3.8$ based on the estimate in [18]. We assume that 30% of individuals work in the same 1km^2 cell where they live, and the rest are distributed according to the gravity model. The appropriate number of workplaces are placed randomly within each 1km^2 cell.

4. Everyone who is not assigned work or school stays home all day.

References

1. R Development Core Team (2011) R: A Language and Environment for Statistical Computing. R Foundation for Statistical Computing, Vienna, Austria. URL `http://www.R-project.org`.

2. Nishiura H, Halstead SB (2007) Natural history of dengue virus DENV-1 and DENV-4 infections: reanalysis of classic studies. J Infect Dis 195: 1007-13.

3. Vaughn DW, Green S, Kalayanarooj S, Innis BL, Nimmannitya S, et al. (1997) Dengue in the early febrile phase: viremia and antibody responses. J Infect Dis 176: 322-30.

4. Egger JR, Coleman PG (2007) Age and clinical dengue illness. Emerg Infect Dis 13: 924-5.

5. Yasuno M, Tonn RJ (1970) A study of biting habits of *Aedes aegypti* in Bangkok, Thailand. Bull World Health Organ 43: 319-25.

6. Styer LM, Carey JR, Wang JL, Scott TW (2007) Mosquitoes do senesce: departure from the paradigm of constant mortality. Am J Trop Med Hyg 76: 111-7.

7. Magori K, Legros M, Puente ME, Focks DA, Scott TW, et al. (2009) Skeeter Buster: A stochastic, spatially explicit modeling tool for studying *Aedes aegypti* population replacement and population suppression strategies. PLoS Negl Trop Dis 3: e508.

8. Harrington LC, Scott TW, Lerdthusnee K, Coleman RC, Costero A, et al. (2005) Dispersal of the dengue vector *Aedes aegypti* within and between rural communities. Am J Trop Med Hyg 72: 209-20.

9. Vaughn DW, Green S, Kalayanarooj S, Innis BL, Nimmannitya S, et al. (2000) Dengue viremia titer, antibody response pattern, and virus serotype correlate with disease severity. J Infect Dis 181: 2-9.

10. Halloran ME, Struchiner CJ, Longini IM Jr (1997) Study designs for evaluating different efficacy and effectiveness aspects of vaccines. Am J Epidemiol 146: 789-803.

11. Halstead SB (2003) Neutralization and antibody-dependent enhancement of dengue viruses. Adv Virus Res 60: 421-67.

12. Fried JR, Gibbons RV, Kalayanarooj S, Thomas SJ, Srikiatkhachorn A, et al. (2010) Serotype-specific differences in the risk of dengue hemorrhagic fever: an analysis of data collected in Bangkok, Thailand from 1994 to 2006. PLoS Negl Trop Dis 4: e617.

13. Guzmán MG, Kouri G, Valdes L, Bravo J, Alvarez M, et al. (2000) Epidemiologic studies on dengue in Santiago de Cuba, 1997. Am J Epidemiol 152: 793-9.

14. Guzmán MG, Kouri G, Bravo J, Valdes L, Vazquez S, et al. (2002) Effect of age on outcome of secondary dengue 2 infections. Int J Infect Dis 6: 118-24.

15. Center for International Earth Science Information Network (CIESIN), Columbia University; International Food Policy Research Institute (IPFRI); the World Bank; and Centro Internacional de Agricultura Tropical (CIAT) (2004). Global rural-urban mapping project, version 1 (GRUMPv1). Available at http://sedac.ciesin.columbia.edu/gpw/.

16. Minnesota Population Center (2011). Integrated public use microdata series, international. Available at https://international.ipums.org/international/.

17. Longini IM Jr, Nizam A, Xu S, Ungchusak K, Hanshaoworakul W, et al. (2005) Containing pandemic influenza at the source. Science 309: 1083-7.

18. Ferguson NM, Cummings DAT, Cauchemez S, Fraser C, Riley S, et al. (2005) Strategies for containing an emerging influenza pandemic in Southeast Asia. Nature 437: 209-14.

19. Viboud C, Bjørnstad ON, Smith DL, Simonsen L, Miller MA, et al. (2006) Synchrony, waves, and spatial hierarchies in the spread of influenza. Science 312: 447-51.
