# Supplementary material for: Controlling Dengue with Vaccines in Thailand
Source: PLoS Negl Trop Dis. 2012 Oct 25;6(10):e1876. doi: 10.1371/journal.pntd.0001876 (PMC3493390; doi:10.1371/journal.pntd.0001876)
Supplement: Text S2 — Parameter selection for the model. (PDF) [file pntd.0001876.s005.pdf]

Online Supporting Text S2: Parameter selection for the model

Dennis L. Chao[1], Scott B. Halstead[2], M. Elizabeth Halloran[1,3], Ira M. Longini, Jr[4,*]
1 Center for Statistics and Quantitative Infectious Diseases, Vaccine and Infectious Disease Division, Fred Hutchinson Cancer Research Center, Seattle, Washington, USA
2 Dengue Vaccine Initiative, Seoul, South Korea
3 Department of Biostatistics, School of Public Health, University of Washington, Seattle, Washington, USA
4 Department of Biostatistics, College of Public Health and Health Professions, and Emerging Pathogens Institute, University of Florida, Gainesville, Florida, USA
∗ E-mail: Corresponding ilongini@ufl.edu

S2 Parameter selection for the model

S2.1 Transmissibility of dengue

We calibrated the model's mosquito-related parameters to match the dynamics of the 1969 DENV-2 outbreak in Puerto Rico described in [1]. This study reported the number of dengue cases per household as well as the household sizes, allowing an accurate estimate of symptomatic attack rates and a qualitative feel for the clustering of cases within individual households, which we presume is partially caused by the behavior of individual infected mosquitoes. [1] estimated the infection attack rate during this outbreak to be 70%, with an apparent:inapparent ratio of 57:43. The epidemic appeared to peak during the week of June 28, and might have started in May or earlier. During the peak, there were about 4–8 illnesses per 100 persons per week.

We ran simulations of communities of 630 immunologically naïve individuals to match the approximate size of the neighborhoods studied in [1]. We populated these communities with synthetic populations of n households using the Ratchaburi household microdata [2]. The communities do not have schools or workplaces (i.e., all people stay home all day). We assumed 20 mosquitoes per household and seeded the epidemic by infecting six individuals (about 1% of the population). We assumed no prior exposure to dengue and an age-dependent symptomatic fraction based on DENV-1 data as described in Section S1.4. About 65% of those infected were symptomatic in our simulations, which is comparable to the 57% estimated by [1].

We performed a grid search to find the values of β_{MP}, β_{PM}, and the daily probability for mosquito migration for the epidemic to peak in 90 days and to have a final infection attack rate of 60% after 150 days. Because the generation interval is long (24 days), we let the runs "burn in" for 48 days (2 generation intervals) to give the mosquitoes time to become infected and disperse. Therefore, we add 48 days to the 6 week target, when the peaks were observed in Puerto Rico. We compared our simulation results to these targets, as shown in Figure S2.1. Runs for which the infection attack rate was between 45% and 75% and peaked between 80 and 100 days:

daily move prob	β_{MP}	β_{PM}	attack rate	peak day
0.1	0.3	0.1	70%	94
0.15	0.25	0.1	61%	92
0.2	0.1	0.25	71%	93
0.35	0.1	0.2	62%	86
0.35	0.4	0.05	67%	91
0.4	0.4	0.05	70%	89
0.45	0.1	0.2	64%	99
0.45	0.2	0.1	66%	99

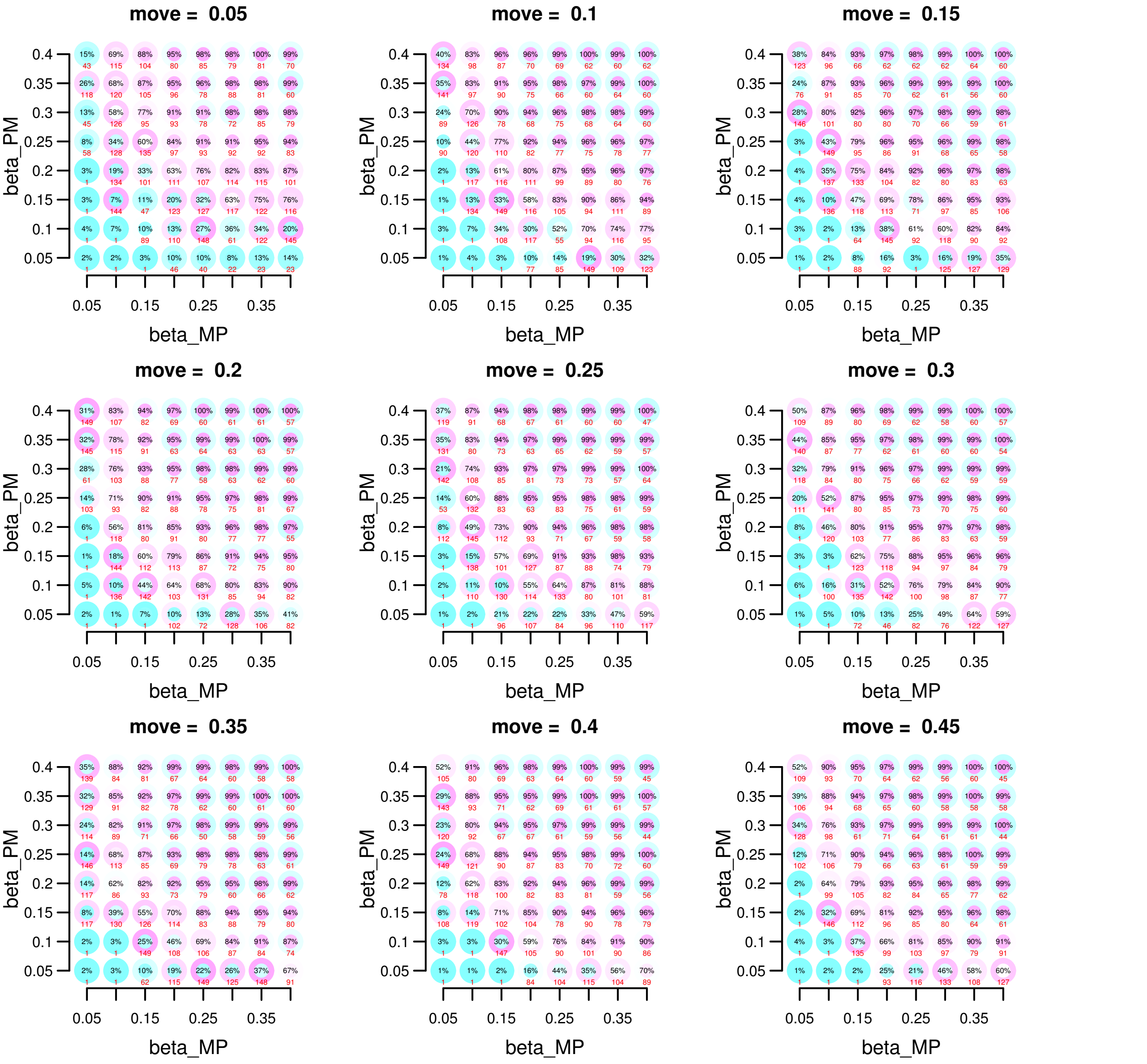

Figure S2.1. Search for reasonable values for β_{MP}, β_{PM}, and the daily probability for mosquito migration. Each sub-plot is for a fixed value for the daily probability of mosquito migration, and the axes represent different values for β_{MP} and β_{PM}. The black numbers are the final infection attack rates (after 150 days) and the red numbers are the day of the epidemic peak, The colors of the inner and outer dots represent how far these values deviate from our target of a 60% attack rate with an epidemic peak at 90 days, with blue for low and pink for high.

In addition to the infection attack rate and the timing of the epidemic peak, we can use the distribution of the numbers of cases per house to calibrate our simulation. [1] tabulates the number of symptomatic cases in each household by household size during a 1969 DENV-2 outbreak in Puerto Rico:

family size	num families	num cases	0	1	2	3	4	5	6	7	8
2	50	21	33	13	4						
3	50	47	24	8	15	3					
4	82	90	36	20	15	4	7				
5	80	122	28	18	16	4	10	4			
6	50	62	21	10	9	6	4	0	0		
7	36	85	11	4	4	9	2	2	0	4	
8	81	192	19	12	11	9	7	3	4	3	6

Expressed as fractions of households of size x with n cases:

family size	0	1	2	3	4	5	6	7	8
2	0.66	0.26	0.08						
3	0.48	0.16	0.30	0.06					
4	0.44	0.24	0.18	0.05	0.09				
5	0.35	0.23	0.20	0.05	0.13	0.05			
6	0.42	0.20	0.18	0.12	0.08	0.00	0.00		
7	0.31	0.11	0.11	0.25	0.06	0.06	0.00	0.11	
8	0.26	0.16	0.15	0.12	0.09	0.04	0.05	0.04	0.08

Comparing our simulation results that produce plausible infection attack rates and epidemic peak timing (Table S2.1) to the data from [1], the best qualitative fit occurs when the mosquito daily migration probability is 15% per day. Thus, reasonable household attack rates and epidemic dynamics are achieved when the mosquito daily migration probability is 15% per day, $\beta_{MP} = 0.25$, and $\beta_{PM} = 0.10$. With these parameters, about 20% of households with 2–7 members have no symptomatic dengue cases. When mosquitoes migrate more often (e.g., 40% probability per day), large households will rarely have no cases because the high dispersal of mosquitoes homogenizes exposure. When mosquitoes migrate rarely (e.g., <10% per day), large households also rarely have no cases because a single infected mosquito will remain in a house and infect many individuals. Higher mosquito migration rates also make the outbreak spread more quickly.

Ideally, we would like to know the number of infected people per household, but only the number of symptomatic cases was ascertained in the Puerto Rican study [1]. A household-based study that ascertains both the number of infections and the number of symptomatic dengue cases in a single dengue season in georeferenced households would help refine our estimates.

Table S2.1. Household infection attack rates in simulations of a small Puerto Rican neighborhood.

move	β_{MP}	β_{PM}	AR	peak	size	N	0	1	2	3	4	5	6	7	8	
0.1	0.3	0.1	70%	94	1	11	0.18	0.82								
					2	27	0.07	0.22	0.7							
					3	28	0.18	0.04	0.14	0.64						
					4	42	0.12	0.1	0.1	0.31	0.38					
					5	29	0.03	0.07	0.14	0.28	0.24	0.24				
					6	12	0	0.17	0.08	0.08	0.25	0.25	0.17			
					7	6	0	0	0.17	0	0.5	0	0.17	0.17		
					8	4	0	0	0	0.25	0	0.25	0	0.25	0.25	
					9	1	0	0	0	0	1	0	0	0	0	0
0.15	0.25	0.1	61%	92	1	1	11	0	1							
					2	27	0.11	0.22	0.67							
					3	28	0.07	0.18	0.14	0.61						
					4	42	0.12	0.05	0.26	0.31	0.26					
					5	29	0.07	0.14	0.24	0.21	0.17	0.17				
					6	12	0.25	0	0.17	0.42	0.08	0.08	0			
					7	6	0.17	0	0.17	0.17	0.17	0.17	0.17	0		
					8	4	0	0	0	0	1	0	0	0	0	
					9	1	0	0	0	1	0	0	0	0	0	0
0.2	0.1	0.25	71%	93	1	11	0	1								
					2	27	0	0.11	0.89							
					3	28	0	0.07	0.43	0.5						
					4	42	0	0	0.24	0.29	0.48					
					5	29	0	0.24	0.14	0.24	0.31	0.07				
					6	12	0	0.08	0.25	0.25	0.17	0.17	0.08			
					7	6	0	0	0.17	0.17	0	0.5	0.17	0		
					8	4	0	0	0.25	0.25	0	0.25	0	0.25	0	
					9	1	0	0	0	0	0	1	0	0	0	0
0.35	0.1	0.2	62%	86	1	11	0	1								
					2	27	0	0.3	0.7							
					3	28	0.07	0.18	0.36	0.39						
					4	42	0.1	0.07	0.24	0.4	0.19					
					5	29	0	0.21	0.28	0.1	0.28	0.14				
					6	12	0	0.17	0.25	0.17	0.08	0.25	0.08			
					7	6	0	0.17	0.17	0	0.5	0.17	0	0		
					8	4	0	0	0.5	0	0.5	0	0	0	0	
					9	1	0	0	0	0	1	0	0	0	0	0
0.35	0.4	0.05	67%	91	1	11	0	1								
					2	27	0.07	0.04	0.89							
					3	28	0.04	0.14	0.18	0.64						
					4	42	0.02	0.1	0.14	0.33	0.4					
					5	29	0.07	0.1	0.21	0.1	0.34	0.17				
					6	12	0.25	0.08	0.25	0.17	0	0.17	0.08			
					7	6	0	0	0.17	0.33	0.17	0.17	0.17	0		
					8	4	0	0	0.5	0	0	0.25	0	0.25	0	
					9	1	0	0	1	0	0	0	0	0	0	0
0.4	0.4	0.05	70%	89	1	11	0.09	0.91								
					2	27	0	0.15	0.85							
					3	28	0.04	0.04	0.29	0.64						
					4	42	0.02	0.1	0.29	0.29	0.31					
					5	29	0.1	0.14	0.1	0.1	0.21	0.34				
					6	12	0.08	0	0.08	0.17	0.25	0.25	0.17			
					7	6	0	0.17	0	0.33	0.17	0.17	0.17	0		
					8	4	0	0	0	0.5	0	0.25	0.25	0	0	
					9	1	0	0	1	0	0	0	0	0	0	0
0.45	0.1	0.2	64%	99	1	11	0	1								
					2	27	0	0.15	0.85							
					3	28	0	0.29	0.25	0.46						
					4	42	0	0.02	0.33	0.38	0.26					
					5	29	0.03	0.14	0.21	0.24	0.21	0.17				
					6	12	0	0.08	0.5	0.33	0	0.08	0			
					7	6	0	0	0.17	0.5	0	0.33	0	0		
					8	4	0	0	0	0.5	0.25	0.25	0	0	0	
					9	1	0	0	0	0	1	0	0	0	0	0
0.45	0.2	0.1	66%	99	1	11	0	1								
					2	27	0	0.22	0.78							
					3	28	0	0.07	0.39	0.54						
					4	42	0.07	0.1	0.24	0.33	0.26					
					5	29	0	0.14	0.21	0.31	0.24	0.1				
					6	12	0.08	0	0.25	0.42	0.08	0	0.17			
					7	6	0	0	0.17	0.17	0.33	0.17	0	0.17		
					8	4	0	0	0.25	0.25	0	0.25	0.25	0	0	
					9	1	0	0	0	1	0	0	0	0	0	0

S2.2 Relative reporting rates of the four dengue serotypes

We assume that the incidence of dengue is the same each year, but the proportion of infections attributed to the four serotypes changes and can be estimated from surveillance data. Because the serotypes have different pathogenicities (Section S1.4), the serotypes would have different reporting rates (with respect to infection). To correct for this, we scale the number of cases of DENV-1, DENV-2, and DENV-3 detected in past years (Tables S1 and S2) by the scalars $d1$, $d2$, and $d3$. DENV-4 cases are not adjusted. We scaled the serotype surveillance data from 1973 to 2008 using various combinations of $d1$, $d2$, and $d3$, used this to construct an serotype-specific exposure history for the synthetic Thai population, and ran our simulation. We compared the number of people symptomatically infected in the simulations to observed relative prevalences of the 4 dengue serotypes in 2009, scaled using $d1$, $d2$, and $d3$ (Figure S2.2). We did not perform the same procedure for years before 2009 because we did not include the modeling mechanisms required to reproduce realistic serotype cycling dynamics [3, 4]. Therefore, we chose to calibrate the model to best fit the most recent publicly available data (i.e., 2009). Because the purpose of this modeling effort is to evaluate the effects of mass vaccination with a highly effective tetravalent vaccine, serotype cycles are not an outcome of interest for this study. In the simulations, there are seasonally varying mosquito populations as described in Section S2.4, $\beta_{PM} = 0.10$, $\beta_{MP} = 0.25$, DENV-2 and DENV-4 are 25% as pathogenic as DENV-1 and DENV-3, pathogenicity of secondary infections is equal to primary, and a 365 day simulation. By inspection, $d1$ should be greater than 0.1 and less than 0.4. A good fit occurs when $d1 = 0.3$, $d2 = 0.2$, and $d3 = 0.5$. The re-scaled exposure history in our model is shown in Figure 3.

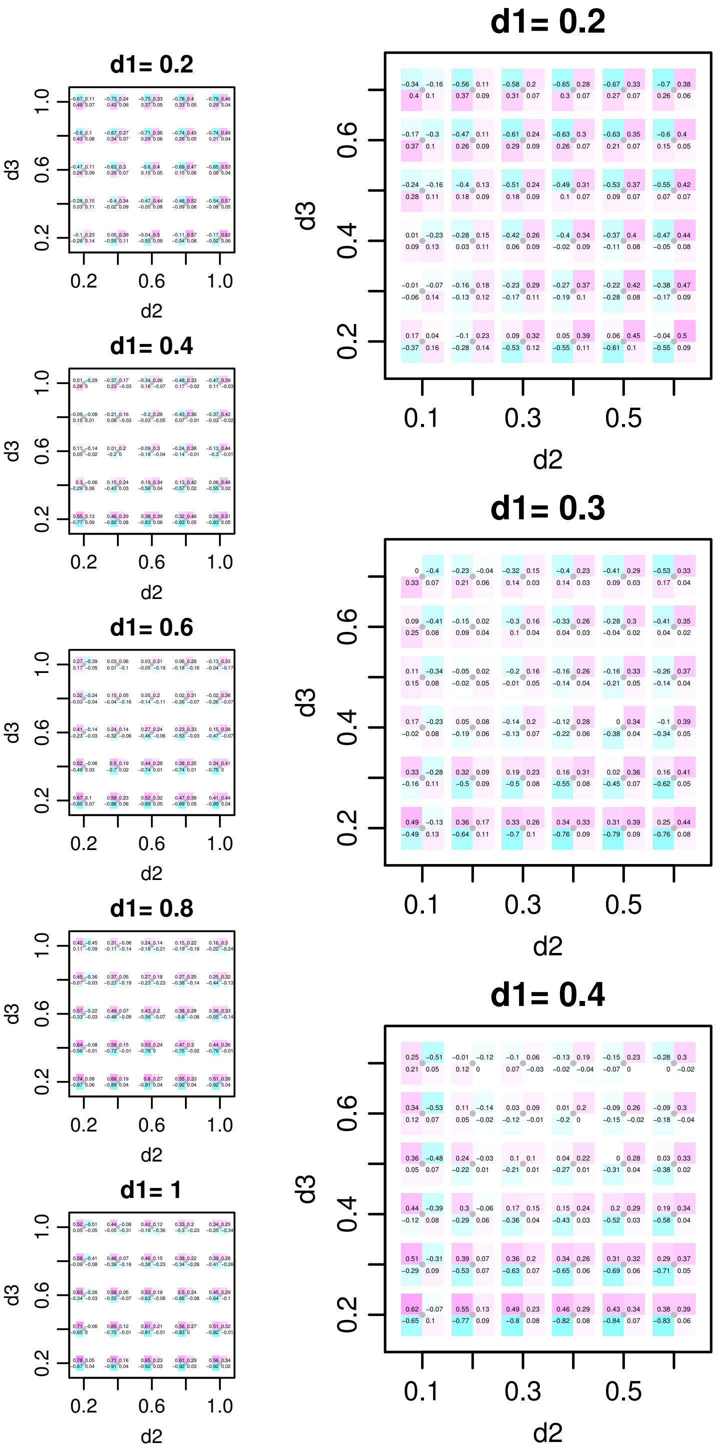

Figure S2.2. Differences between 2009 serotype surveillance data and the simulation. The surveillance data for DENV-1, DENV-2, and DENV-3 were scaled by $d1$, $d2$, and $d3$, respectively. We used various values of these scaling factors, as shown in the five plots. The numbers to the upper left of each dot are the absolute difference between observed and simulated relative prevalence of DENV-1, with DENV-2 being at the upper right, DENV-3 being at the lower left, and DENV-4 being at the lower right. The color reflects the deviation from 0.

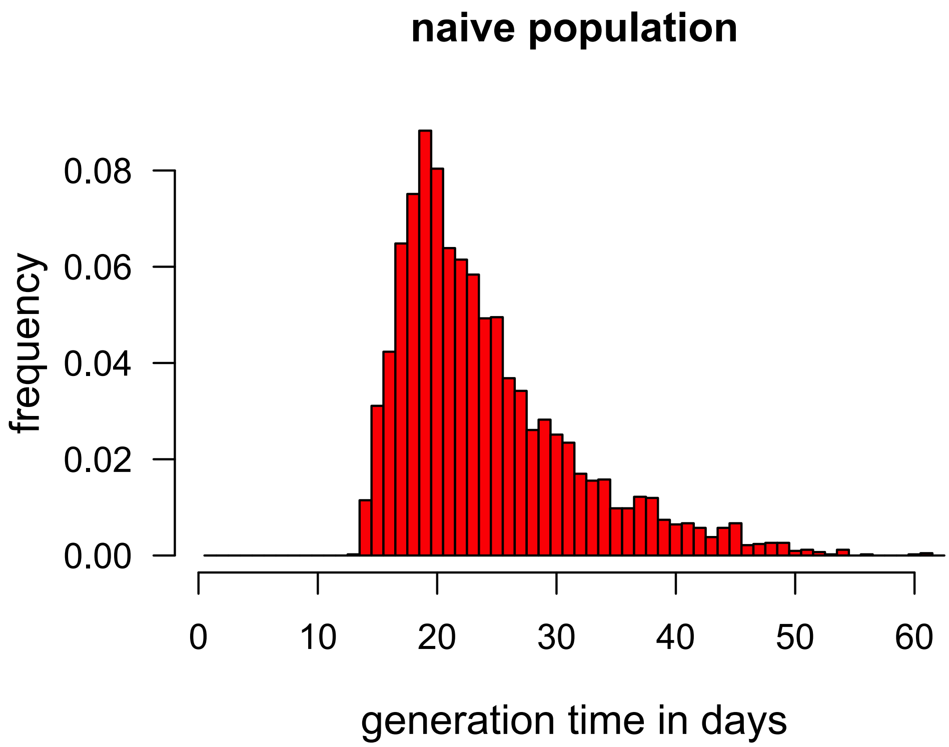

Figure S2.3. Generation time in an immunologically naïve Bang Phae population. We infected a single person, and recorded the infection time of the subsequent infected individuals, who were not infectious in these simulations. The procedure was repeated 200 times per serotype, and the results from all runs were combined.

S2.3 Estimating the generation interval and R_0

We calculate crude R_0 estimates by infecting a single randomly-chosen individual with one of the four serotypes then running a simulation for 70 days with no secondary transmission (i.e., only the index case is infectious). We assumed that each building had 42 mosquitoes, corresponding to our estimate of the peak of dengue transmission in Thailand. We find that the median generation time is 22.0 days and the mean is 24.1 days (Figure S2.3). This procedure will underestimate R_0 because we randomly select the primary case uniformly across the entire population, rather than giving those most likely to transmit more weight. Running the simulation 200 times for each serotype, we find that for an immunologically naïve population, there are an average of 5.30 (range of 0–29) infections for DENV-1 and DENV-3 and 5.16 (range of 0–24) infections for DENV-2 and DENV-4 (Figure S2.4). We assumed that DENV-2 and DENV-4 were 25% as pathogenic as DENV-1 and DENV-3. Although symptomatic and asymptomatic individuals are equally infectious, symptomatic people may stay home due to illness (Section S1.3), which lowers the probability of infecting mosquitoes outside the household. For the population with the estimated Thai exposure to dengue, we get an average of 2.34, 2.08, 2.10, and 1.85 secondary infections for each of the four serotypes.

Various estimates of R for dengue from the modeling literature are listed below:

R_0	*Setting*	*Method*	*Reference*
1.33–2.41	Mexico	fraction infected	[5]
1.9	—	derived from model	[6]
1.6–2.4	Brazil		[7]
4.29–5.75	Thailand	model fit	[8]
2.0–3.3–103	Brazil	fit simple ODE model	[9]
0.49–3.30	Mexico	fit big ODE model	[10]
2.3–11	Brazil	exponential fit	[11]
5.2–6.7	Thailand	model fit	[12]
0.28–5.04	Brazil	Ross Macdonald model fit	[13]

In our model, we expect R_0 to be proportional to the vector population as it is in the Ross–Macdonald model [14]:

$$R_0 = \frac{ac}{r} \times \frac{mabe^{-\mu\tau}}{\mu} = \frac{ma^2bce^{-\mu\tau}}{r\mu} \tag{1}$$

Figure S2.5 plots transmissibility vs the number of (implicit susceptible) mosquitoes per location. Note that R_0 is approximately 1.0 when there are 20 mosquitoes per location (Figure S2.5B). Thus, smaller mosquito populations would not sustain dengue transmission.

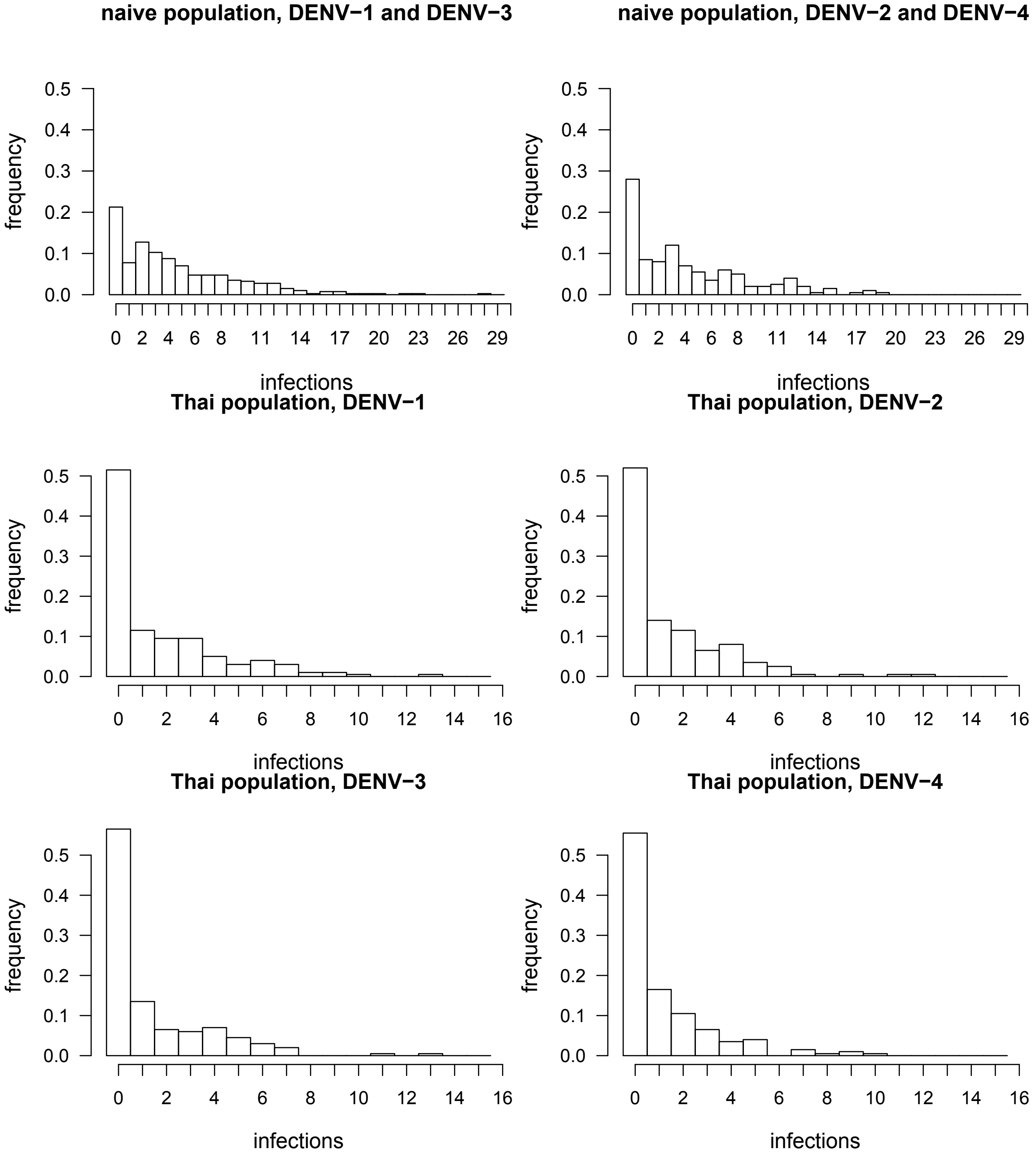

Figure S2.4. Number of secondary dengue infections per infected individual. A single randomly selected individual is infected and the number of secondary infections is tallied. Top two panels, which use immunologically naïve populations, show the distribution of the number of secondary cases (200 runs per serotype) and are crude estimates of R_0. The remaining panels are the distributions of secondary cases of each of the four dengue serotypes in a population with the estimated Thai exposure to dengue (200 runs per serotype).

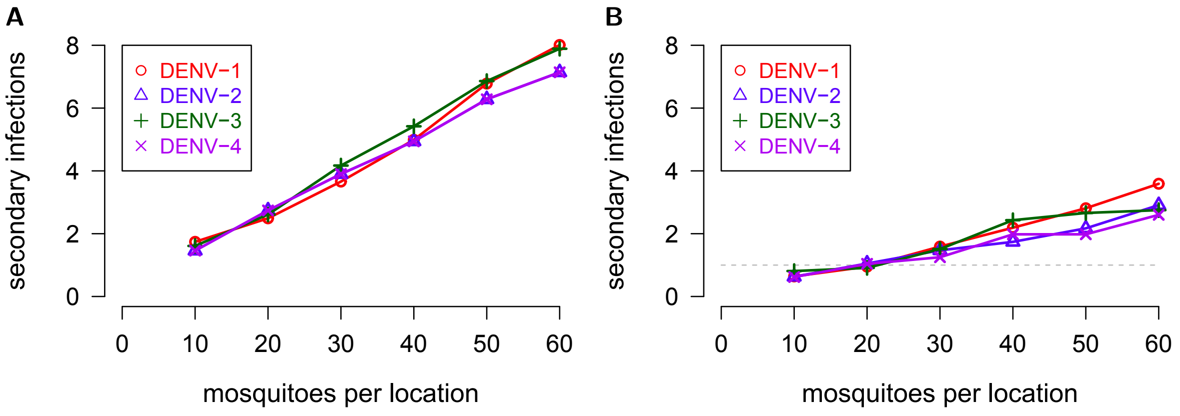

Figure S2.5. Estimates of dengue transmissibility vs the mosquito population size per location. (A) The number of infections generated by a single infected person in a naïve population. Points are the means from 100 simulation runs (using randomly selected index cases). (B) The number of infections generated by a single infected person in a population with the estimated immune history of Bang Phae.

S2.4 Seasonality of dengue

We assume that changes in mosquito population size drive dengue seasonality. In Section S2.4, we observe that having 20 mosquitoes per location can sustain dengue transmission, while lower and higher numbers will decrease or increase dengue prevalence. Mosquito counts peak in May–June (Figure S2.6). It has been observed that DHF activity lags mosquito counts by 2 months [15].

If we set the maximum mosquito capacity to 42, and for each serotype expose 2 individuals each day (total of 8 people) throughout a 364-day run, we get an infection attack rate of 11% among dengue-naïve individuals and an epidemic peak in July–August (Figure S2.6).

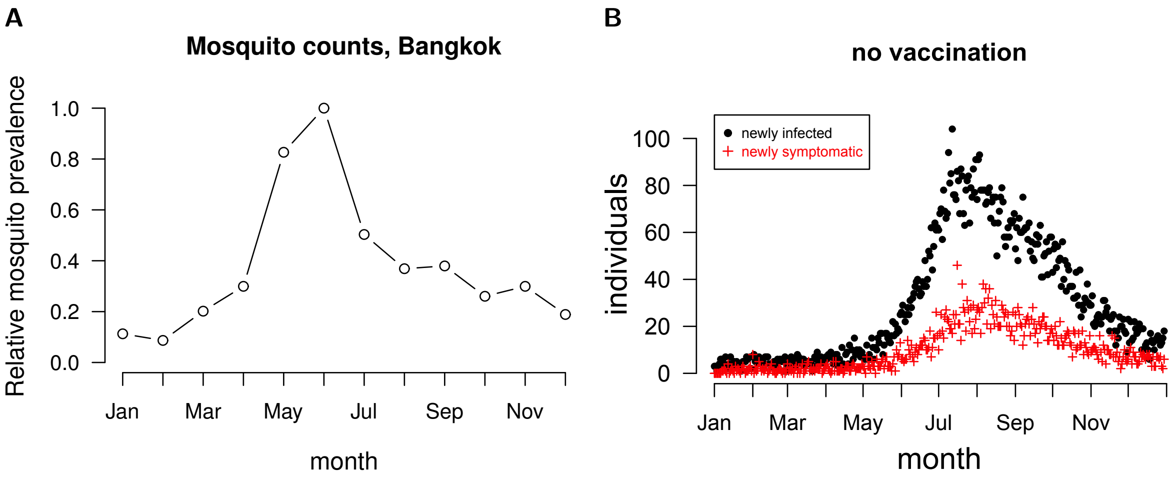

Figure S2.6. Mosquito counts and seasonality. (A) Relative number of mosquitoes by month in Bangkok. We use a step function with a one-week temporal resolution to match the observations in [15]. (B) A simulation using this mosquito population seasonality.

References

1. Likosky WH, Calisher CH, Michelson AL, Correa-Coronas R, Henderson BE, et al. (1973) An epidemiologic study of dengue type 2 in Puerto Rico, 1969. Am J Epidemiol 97: 264-75.

2. Minnesota Population Center (2011). Integrated public use microdata series, international. Available at https://international.ipums.org/international/.

3. Adams B, Holmes EC, Zhang C, Mammen MP Jr, Nimmannitya S, et al. (2006) Cross-protective immunity can account for the alternating epidemic pattern of dengue virus serotypes circulating in Bangkok. Proc Natl Acad Sci U S A 103: 14234-9.

4. Johansson MA, Hombach J, Cummings DAT (2011) Models of the impact of dengue vaccines: A review of current research and potential approaches. Vaccine 29: 5860-8.

5. Koopman JS, Prevots DR, Marin VMA, Dantes GH, Aquino ZML, et al. (1991) Determinants and predictors of dengue infection in Mexico. Am J Epidemiol 133: 1168-78.

6. Newton EAC, Reiter P (1992) A model of the transmission of dengue fever with an evaluation of the impact of ultra-low volume (ULV) insecticide applications on dengue epidemics. Am J Trop Med Hyg 47: 709-20.

7. Marques CA, Forattini OP, Massad E (1994) The basic reproduction number for dengue fever in Sao Paulo state, Brazil: 1990–1991 epidemic. Trans R Soc Trop Med Hyg 88: 58-9.

8. Ferguson NM, Donnelly CA, Anderson RM (1999) Transmission dynamics and epidemiology of dengue: insights from age-stratified sero-prevalence surveys. Philos Trans R Soc Lond B Biol Sci 354: 757-68.

9. Favier C, Degallier N, Rosa-Freitas MG, Boulanger JP, Lima CJR, et al. (2006) Early determination of the reproductive number for vector-borne diseases: the case of dengue in Brazil. Trop Med Int Health 11: 332-40.

10. Chowell G, Diaz-Dueñas P, Miller JC, Alcazar-Velazco A, Hyman JM, et al. (2007) Estimation of the reproduction number of dengue fever from spatial epidemic data. Math Biosci 208: 571-89.

11. Coelho GE, Burattini MN, da Glória Teixeira M, Coutinho FAB, Massad E (2008) Dynamics of the 2006/2007 dengue outbreak in Brazil. Mem Inst Oswaldo Cruz 103: 535-9.

12. Cummings DAT, Iamsirithaworn S, Lessler JT, McDermott A, Prasanthong R, et al. (2009) The impact of the demographic transition on dengue in Thailand: insights from a statistical analysis and mathematical modeling. PLoS Med 6: e1000139.

13. Massad E, Coutinho FAB, Burattini MN, Amaku M (2010) Estimation of R_0 from the initial phase of an outbreak of a vector-borne infection. Trop Med Int Health 15: 120-6.

14. Macdonald G (1957) The epidemiology and control of malaria. Oxford, United Kingdom: Oxford University Press.

15. Halstead SB (2008) Dengue virus–mosquito interactions. Annu Rev Entomol 53: 273-91.
