# Supplementary material for: Controlling Dengue with Vaccines in Thailand
Source: PLoS Negl Trop Dis. 2012 Oct 25;6(10):e1876. doi: 10.1371/journal.pntd.0001876 (PMC3493390; doi:10.1371/journal.pntd.0001876)
Supplement: Text S3 — Sensitivity of attack rates to maximum infection parity assumptions. (PDF) [file pntd.0001876.s006.pdf]

Online supporting text S3:
Sensitivity of attack rates to maximum infection parity assumptions

Dennis L. Chao[1], Scott B. Halstead[2], M. Elizabeth Halloran[1,3], Ira M. Longini, Jr[4,*]
1 Center for Statistics and Quantitative Infectious Diseases, Vaccine and Infectious Disease Division, Fred Hutchinson Cancer Research Center, Seattle, Washington, USA
2 Dengue Vaccine Initiative, Seoul, South Korea
3 Department of Biostatistics, School of Public Health, University of Washington, Seattle, Washington, USA
4 Department of Biostatistics, College of Public Health and Health Professions, and Emerging Pathogens Institute, University of Florida, Gainesville, Florida, USA
∗ E-mail: Corresponding ilongini@ufl.edu

S3 Sensitivity of attack rates to maximum infection parity assumptions

Prior exposure to more than one serotype could grant protective cross-immunity against infection by the remaining serotypes. One can test this hypothesis by comparing the model's behavior when an individual can be infected by a maximum of 2, 3, or 4 serotypes. This would correspond to perfect protection against infection after the maximum infection parity is reached by an individual. Results in the main text assumed that one could be infected by all 4 serotypes.

The model is sensitive to the maximum infection parity allowed, with substantially lower attack rates when individuals could not be infected by all four serotypes (Figure S3.1). When an individual can only be infected by two or three serotypes, transmissibility must be increased to produce comparable overall infection attack rates, which we did not do here. Another consequence of limiting the maximum infection parity is a shift in the age distribution of infections and symptomatic cases. Figure S3.2A, like Figure 5, plots the age distribution of infections and uncomplicated dengue fever cases when individuals can be infected by all four serotypes (maximum infection parity of four). As the maximum infection parity is limited to three (Figure S3.2B) and two (Figure S3.2C), contribution of adults to the numbers of infections and cases is reduced.

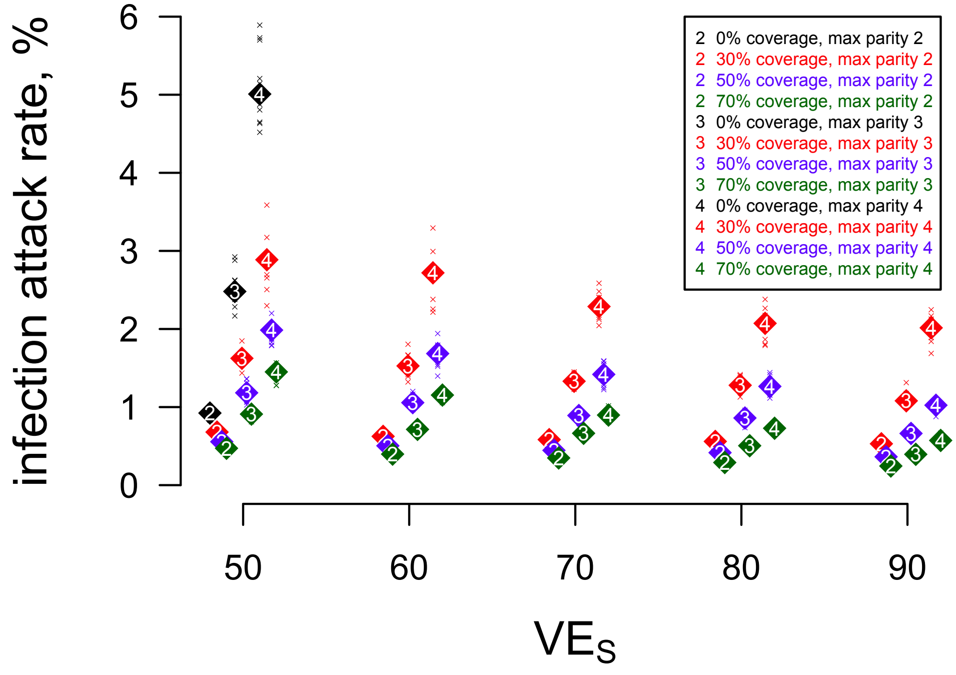

Figure S3.1. Sensitivity of mass vaccination effectiveness to maximum infection parity. We ran the model assuming different maximum infection parities (2, 3, or 4). We ran the simulation ten times for each combination of parameters. Infection attack rates for different vaccine efficacies and coverage levels. We varied the vaccine efficacy (VE_S=50%, 60%, 70%, 80%, and 90% as indicated on the x-axis) and coverages of individuals ages 2–46 (0%, 30%, 50%, and 70% shown in black, red, blue, and green, respectively). Diamonds indicate the median infection attack rates, and each X is the rate from a single stochastic simulation.

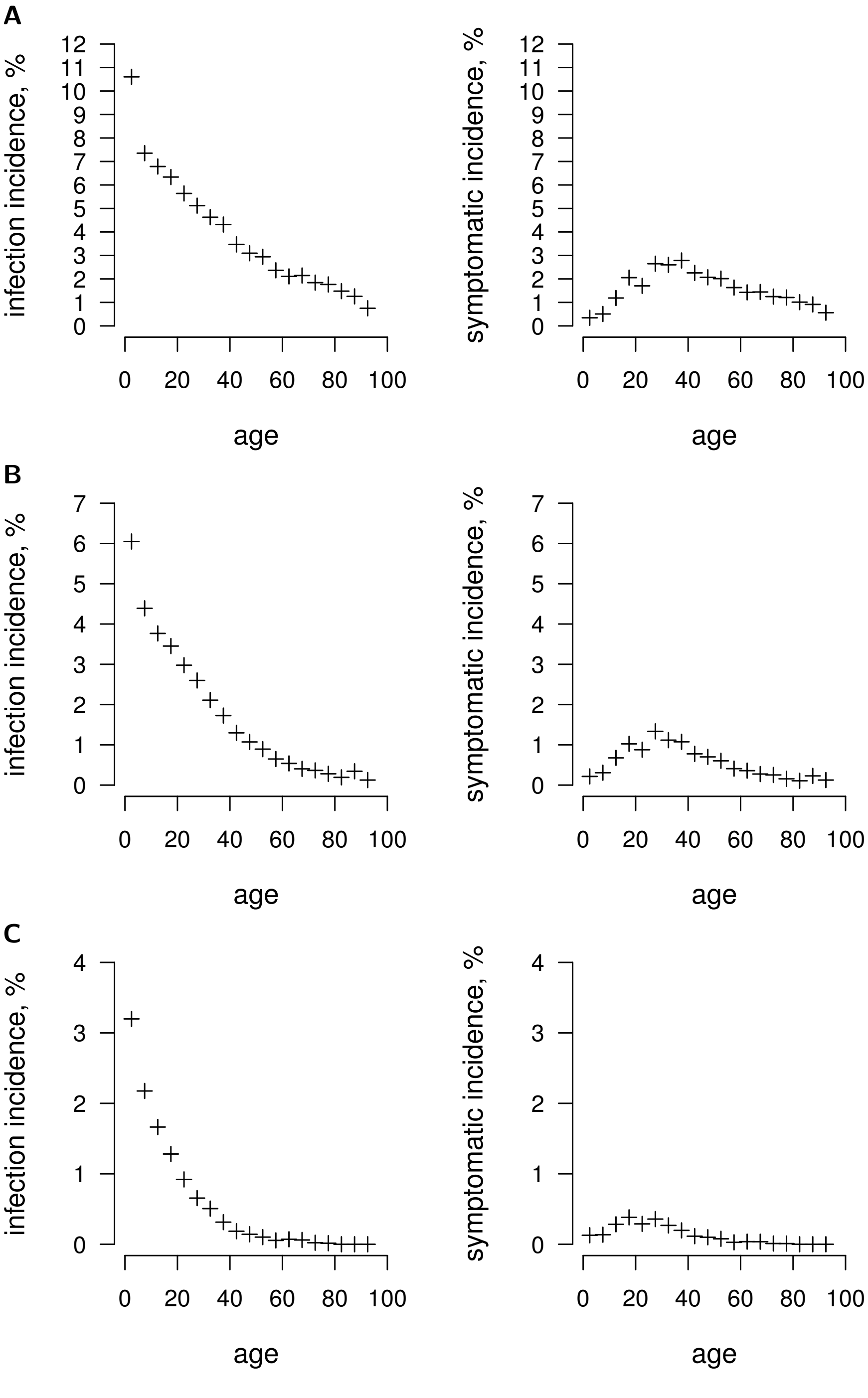

Figure S3.2. Sensitivity of attack rates to maximum infection parity. Infection and symptomatic attack rates by age with no vaccination. We ran the model assuming different maximum infection parities (2, 3, or 4). We ran the simulation ten times for each combination of parameters. Each point represents a 5-year age bracket as in Figure 5. An individual may be sequentially infected by (A) all four serotypes, (B) three serotypes, and (C) two serotypes.
