# Supplementary material for: Controlling Dengue with Vaccines in Thailand
Source: PLoS Negl Trop Dis. 2012 Oct 25;6(10):e1876. doi: 10.1371/journal.pntd.0001876 (PMC3493390; doi:10.1371/journal.pntd.0001876)
Supplement: Text S4 — Sensitivity of attack rates to vaccine efficacy. (PDF) [file pntd.0001876.s007.pdf]

Online Supporting Text S4:
Sensitivity of attack rates to vaccine efficacy

Dennis L. Chao[1], Scott B. Halstead[2], M. Elizabeth Halloran[1,3], Ira M. Longini, Jr[4,*]
1 Center for Statistics and Quantitative Infectious Diseases, Vaccine and Infectious Disease Division, Fred Hutchinson Cancer Research Center, Seattle, Washington, USA
2 Dengue Vaccine Initiative, Seoul, South Korea
3 Department of Biostatistics, School of Public Health, University of Washington, Seattle, Washington, USA
4 Department of Biostatistics, College of Public Health and Health Professions, and Emerging Pathogens Institute, University of Florida, Gainesville, Florida, USA
∗ **E-mail: Corresponding ilongini@ufl.edu**

S4 Sensitivity of attack rates to vaccine efficacy

To test the sensitivity of the results to our vaccine efficacy assumptions, we ran the model for several different values of $\mathsf{VE_S}$ using different pre-vaccination coverage levels and different assumptions of the vaccine's efficacy to lower the probability of symptomatic disease given infection and of infectiousness. For simulations with $\mathsf{VE_S}$ less than 70%, average and variance of infection attack rates rise, as one would expect (Figure S4.1). Increasing efficacy beyond 70% lowers the average attack rate, but with less effects on variance. We also ran simulations in which the the vaccine conferred protection against illness given infection ($\mathsf{VE_P}$) and reduced infectiousness ($\mathsf{VE_I}$) for those not protected from infection ($\mathsf{VE_S}$). Once the vaccine protects over 70% from infection ($\mathsf{VE_S} > 0.7$), reducing the probability of illness or infectiousness upon infection of those vaccinated did not qualitatively affect results (Figure S4.1).

Because a tetravalent vaccine might not be equally protective against all four serotypes, we modeled the effect of vaccines that only protected against infection for three of the four serotypes (equally well) but offered no protection against the remaining serotype. As expected, a vaccine that protects against all four serotypes reduces the infection attack rates more than one that protects against only three (Figure S4.2). The effect appears to be most pronounced for DENV-2 and the least pronounced for DENV-4. When the transmission of only three serotypes is reduced by mass vaccination, the prevalence of the remaining serotype could increase because of the lack of competing co-circulating serotypes.

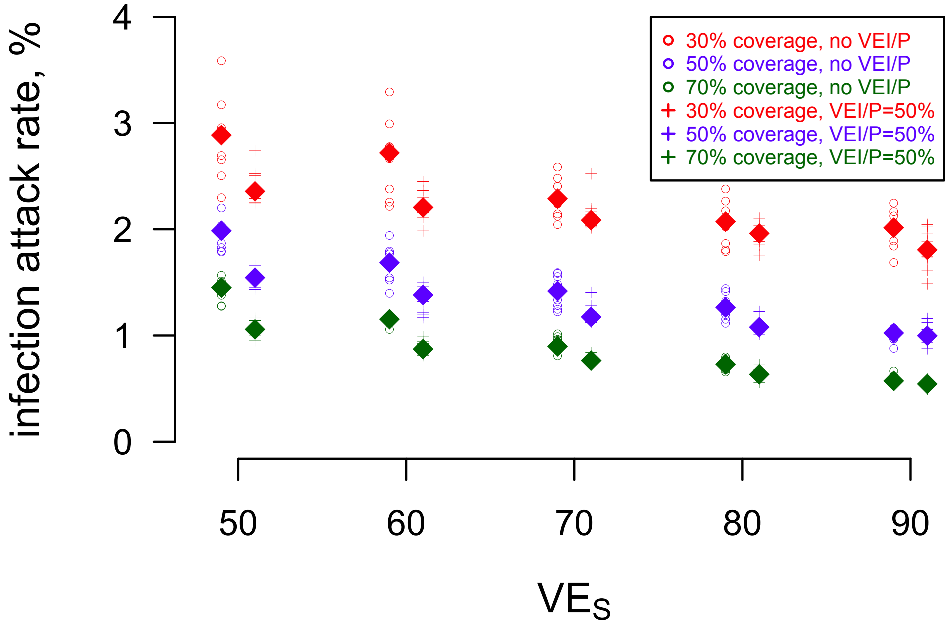

Figure S4.1. Sensitivity of infection attack rates to vaccine efficacy for a single season. The model was run for three different pre-vaccination coverage levels (30%, 50%, and 70% of those aged 2 to 46 years), five different VE_S levels (50%, 60%, 70%, 80%, and 90%), and two different VE_P and VE_I levels (both were set to either 0% or 50%). The diamonds indicate the median infection attack rates for 10 stochastic simulations, and the individual o's and +'s represents the result from a single simulation.

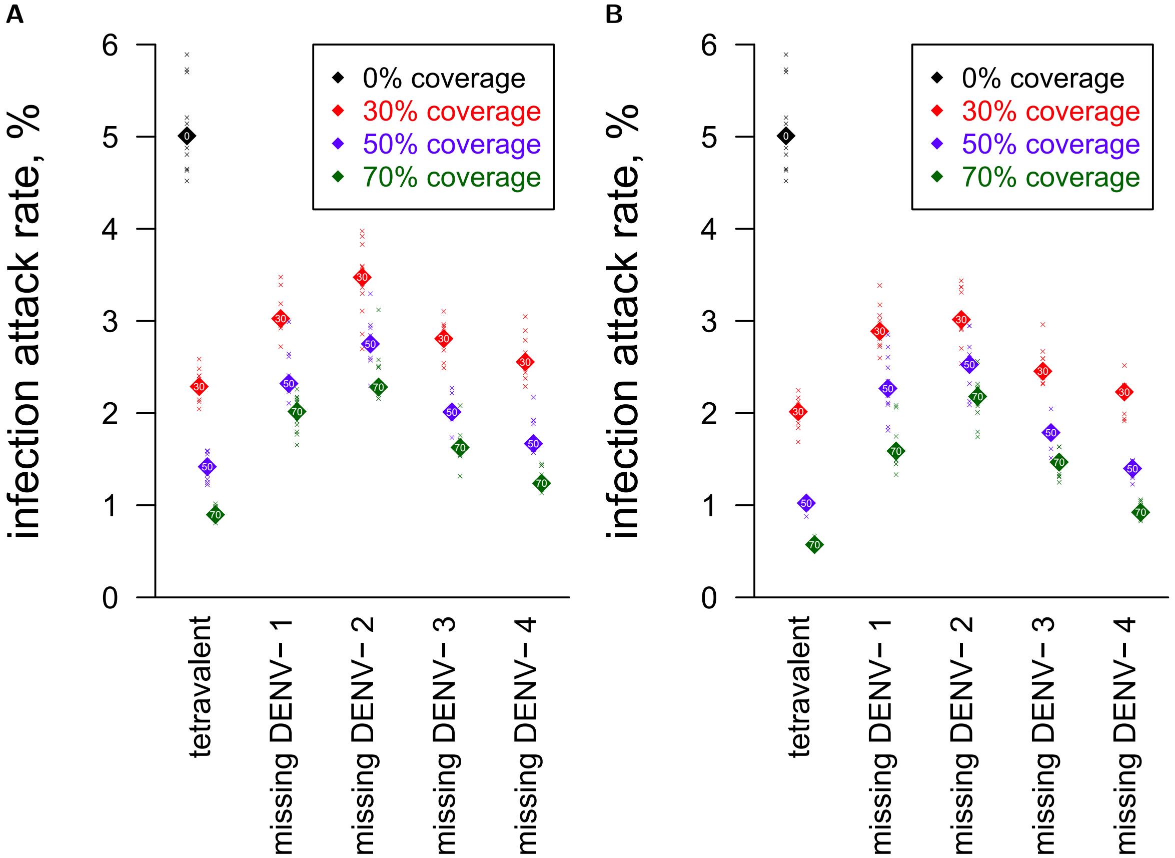

Figure S4.2. Single-season infection attack rates when the vaccine protects against only three of the four serotypes. In the simulations, the vaccine was either equally protective against all four serotypes (i.e., tetravalent) or equally protective against only three serotypes and not effective against the fourth (i.e., missing one of the four serotypes). Simulations were run for four different pre-vaccination coverage levels (0%, 30%, 50%, and 70% of those aged 2 to 46 years, and shown in black, red, blue, and green, respectively) and for (A) VE_S of 70% and (B) VE_S of 90%. Diamonds in the plot represent the median infection attack rates of 10 stochastic simulations for each of the scenarios, and the small Xs are the results of each of the simulations.
